# Systematic Review and Meta-analysis of Group B *Streptococcus* Rectovaginal Colonization Rates and Serotype Distribution among Pregnant Women, Asia-Pacific Region, 2014–2025 

**DOI:** 10.3201/eid3210.260687

**Published:** 2026-10

**Authors:** Zheng Quan Toh, Lien Anh Ha Do, Darren Suryawijaya Ong, Claire von Mollendorf, Fiona M. Russell

**Affiliations:** Murdoch Children’s Research Institute and University of Melbourne, Parkville, Victoria, Australia

**Keywords:** group B *Streptococcus*, systematic review, meta-analysis, pregnant women, colonization, serotype, intrapartum antibiotic prophylaxis, antimicrobial resistance, bacteria, Asia-Pacific region

## Abstract

We conducted a systematic review and meta-analysis of group B *Streptococcus* (GBS) rectovaginal colonization prevalence, serotype distribution, and antimicrobial drug resistance (AMR) rates among pregnant women in the Asia-Pacific region. We included articles published in the MEDLINE, Embase, and PubMed databases during January 2014–October 2025. Overall, 1,623 studies were identified; 98 of those were included in the meta-analysis. Of 978,150 pregnant women, 172,973 were colonized with GBS; pooled estimated prevalence was 18.0% (95% CI 12%–23%). Studies from Southeast Asia were limited. GBS serotype distributions were reported in 24 studies; serotype III was the most common (range 5.0%–100.0%). Thirty studies included AMR rates; low (0%–2.0%) AMR rates were found for β-lactams. GBS colonization is common among pregnant women in the Asia-Pacific region. Continued surveillance of GBS prevalence will be needed, particularly in Pacific and Southeast Asian countries, to guide effective GBS prevention and treatment programs.

Group B *Streptococcus* (GBS), also known as *Streptococcus agalactiae*, is a gram-positive bacterium that causes substantial infant illness and death worldwide (*1*). Approximately 400,000 cases of neonatal invasive GBS disease and >90,000 deaths associated with GBS infections occur globally each year (*2*–*4*). Most GBS cases and associated deaths occur in low- and middle-income countries, where access to antenatal screening and treatment is limited (*2*–*4*).

Maternal rectovaginal GBS colonization is a major risk factor for invasive GBS disease in pregnant women and neonates (*5*). Pregnant women who have rectovaginal GBS colonization can transfer bacteria to their fetuses or newborn infants during delivery, causing early onset of neonatal invasive GBS disease. To reduce GBS transmission, most high-income countries offer intrapartum antibiotic prophylaxis (IAP) to GBS-positive pregnant women, screened at 35–37 weeks’ gestation, or to women who have established risk factors for GBS colonization. Risk factors include prolonged rupture of amniotic membranes, GBS bacteriuria during pregnancy, intrapartum maternal fever, and having previously delivered an infant who had early-onset GBS disease (*6*). Penicillin or ampicillin is the first-line antimicrobial treatment for GBS infection; clindamycin or vancomycin is used for persons who are allergic to penicillin (*7*). However, low-income countries face major challenges in implementing GBS screening and IAP, including lack of national GBS screening guidelines and programs, limited access to prenatal care, lack of laboratory resources for testing, and high prevalences of home births (*8*,*9*). Furthermore, frequent use of antimicrobial drugs has raised concerns regarding the emergence of antimicrobial drug resistance (AMR) (*10*).

Systematic reviews of global maternal rectovaginal GBS colonization up to January 2020 reported the average colonization rate to be 14%–18%; the highest prevalences occurred in Africa and central and South Asia (*2*,*11*,*12*). Those reviews contained limited data from the Asia-Pacific region, particularly the Oceania subregion (*2*,*11*,*12*). Maternal GBS colonization rates were used to estimate global and regional prevalence of invasive GBS in infants; estimates for the Oceania subregion were extrapolated from other regional data (*2*). Among the 10 GBS serotypes, Ia, III, and V were the most common serotypes found in maternal colonization and accounted for >60% of invasive GBS disease in infants (*13*), although serotype distribution varied by region. Serotypes VI–IX were more common in Asia (6%–16%) and West Africa (15%) than in Europe (<2%) or North America (<1%) (*11*,*13*). To inform public health policy and develop preventive and treatment strategies against invasive GBS, we conducted a systematic review and meta-analysis to assess maternal rectovaginal GBS colonization rates, serotype distribution, and AMR rates among pregnant women in the Asia-Pacific region.

## Methods

We registered our systematic review protocol at PROSPERO (https://www.crd.york.ac.uk; registration no. CRD42024535368). We followed the PRISMA (Preferred Reporting Items for Systematic reviews and Meta-Analyses) 2020 guidelines (https://www.prisma-statement.org) for reporting systematic review data.

### Search Strategy and Selection Criteria

We included articles published during January 1, 2014, to October 31, 2025, in MEDLINE (https://www.nlm.nih.gov/medline) and Embase (https://www.embase.com) using the OVID platform (https://www.ovid.com). We used Medical Subject Headings and thesaurus terms and keywords. We searched PubMed (https://pubmed.ncbi.nlm.nih.gov) for articles not yet indexed. We used search terms related to *Streptococcus agalactiae*, carriage, mothers, pregnancy, and Asia-Pacific. We limited our search to studies published in English (Appendix). We included studies reporting GBS colonization rates in pregnant and postpartum women and those reporting samples obtained from vaginal, rectal, or perianal regions. We included all observational and case studies reporting GBS prevalence in pregnant women from East Asia, South Asia, Southeast Asia, and Oceania.

We excluded studies of pregnant women when GBS prevalence estimates were derived from <50 women or when rates could not be ascertained from laboratory sample data. We also excluded modeling, ecologic, and qualitative studies; systematic reviews; opinion-based pieces; and animal studies.

We uploaded the search results to Covidence systematic review management software (https://www.covidence.org) for screening. Two authors independently screened the titles and abstracts of the studies; Z.Q.T. screened 100% and L.A.H.D. screened 10% of randomly selected papers. We excluded duplicate articles during the Covidence uploading and screening stages. Z.Q.T. and L.A.H.D. each independently performed 100% of full-text study screening. The 2 reviewers resolved any disagreements at the screening or full-text stages through mutual consensus.

### Data Extraction and Quality Assessment

One reviewer (Z.Q.T.) extracted data by using Covidence and Microsoft Excel (https://www.microsoft.com) tables. Extracted data included country, study period, study design, study population, gestational age at time of sampling, number of pregnant women tested, number of women with detected GBS, method of detection (culture or molecular, with or without enrichment), sampling site (vagina, rectum, or perianal region at any time during pregnancy), serotype distribution, serotyping methods, AMR rates, and AMR testing method.

We used the Joanna Briggs Institute Critical Appraisal Tool (https://jbi.global/critical-appraisal-tools) for analytical cross-sectional studies to assess the risk of bias scores in the studies; Z.Q.T. performed the assessment. We selected that risk of bias tool because our review focused on the prevalence of GBS colonization, and most studies were observational. We addressed 8 criteria (Appendix Table 1). A yes response received a score of 1, whereas a no or unclear response received a score of 0. We considered a total score of 6–8 as low risk, 3–5 as moderate risk, and 0–2 as high risk for bias.

### Data Analysis

We generated data by using Microsoft Excel and analyzed those data by using Stata version 19.0 (StataCorp LLC, https://www.stata.com). We excluded studies from the meta-analysis that had a high risk for bias (score <3). We pooled GBS prevalences by using a random-effects inverse variance model, weighted by sample size. This model assumed that studies included in the meta-analysis were a random sample from a larger population of studies and that prevalences varied across those studies. We pooled GBS prevalences according to country income levels by using 2026 World Bank country income classifications (https://datahelpdesk.worldbank.org/knowledgebase/articles/906519-world-bank-country-and-lending-groups) and visualized those data on Forrest plots and line graphs; we expressed prevalence as percentage (95% CI). We performed analyses for East Asia, South Asia, Southeast Asia, and Oceania subgroups. We assessed heterogeneity between studies by using an *I*^2^ test. We summarized GBS serotype distribution and AMR rates for studies that reported those outcomes. We generated figures for GBS colonization prevalence rates according to detection methods and serotype distribution, as well as descriptive statistics by using GraphPad Prism version 10 (GraphPad Software, Inc., https://www.graphpad.com).

## Results

### Study Characteristics

We identified 1,623 studies from the literature search; 106 of those met the inclusion criteria (Figure 1). The interrater agreements were 83.4% for title and abstract screening and 88.2% for full-text reviews.

**Figure 1:**
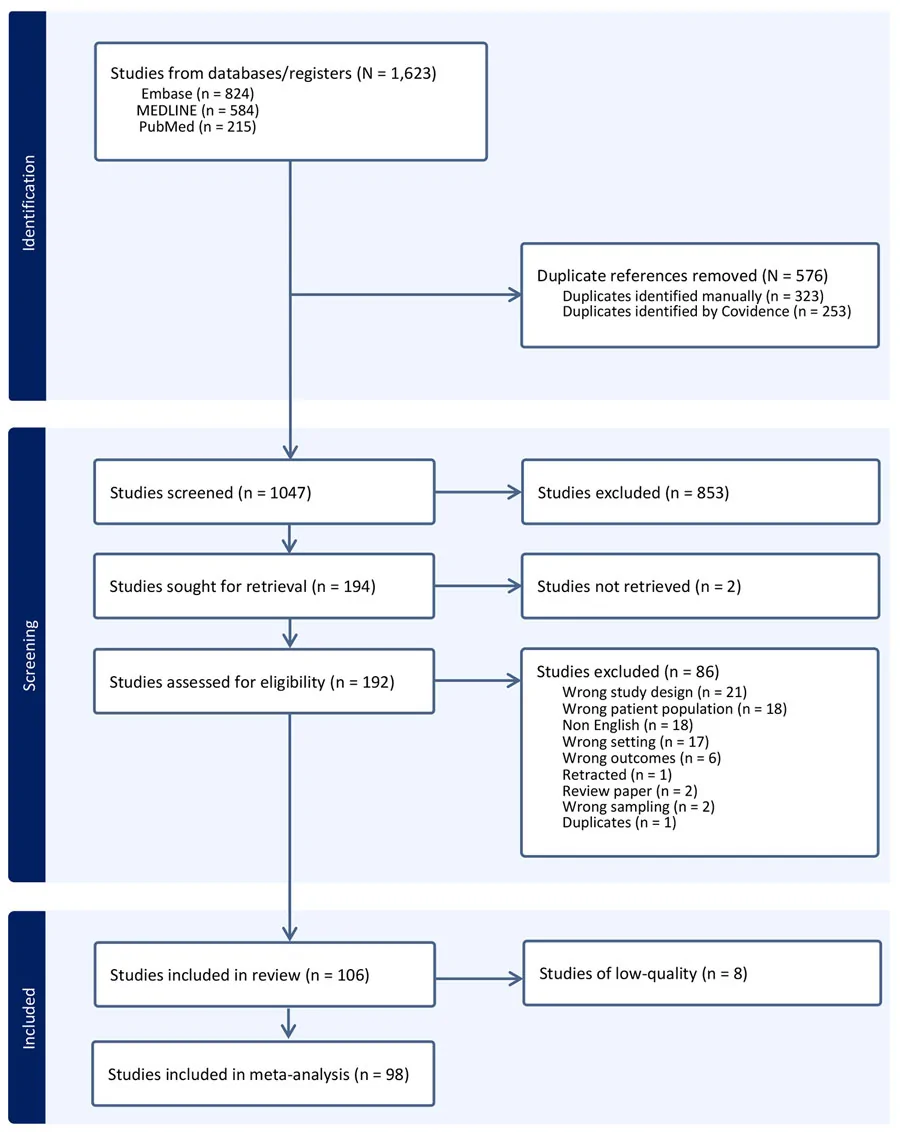
Flow diagram of selection criteria in study of rectovaginal colonization rates and serotype distribution among pregnant women, Asia-Pacific region, 2014–2025. We followed the 2020 Preferred Reporting Items for Systematic reviews and Meta-Analyses guidelines (PRISMA; https://www.prisma-statement.org) and used MEDLINE (https://www.nlm.nih.gov/medline), Embase (https://www.embase.com), and PubMed (https://pubmed.ncbi.nlm.nih.gov) databases to identify included studies. Covidence systematic review management software (https://www.covidence.org) was used to screen and identify duplicate studies.

We summarized study details and participant characteristics for the 106 included studies (Table 1; Appendix Tables 2, 3). Of those studies, 42.5% (45/106) were cross-sectional, 28.3% (30/106) were prospective cohort, and 28.3% (30/106) were retrospective studies; 1 was a case-control study (Appendix Table 3). We found 38 studies reported data from high-income countries (HICs), 38 from upper-middle–income countries (UMICs), and 30 from low-middle–income countries (LMICs) (Table 1); 1 study was a multi-country study that included data from both Bangladesh and India (Appendix Table 3). The studies reported data from a total of 13 countries; 33 (31.1%) studies reported data from China. Most (62 [58.5%]) studies reported data from East Asia, whereas 24 (22.4%) reported data from South Asia, 11 (10.3%) from Southeast Asia, and 10 (9.3%) from Oceania (all from Australia). A total of 25 (23.5%) studies reported serotype distribution, and 34 (32.1%) reported AMR rates.

**Table 1:** Summary of included studies, according to country-income status, in study of group B *Streptococcus* rectovaginal colonization rates and serotype distribution among pregnant women, Asia-Pacific region, 2014–2025*

Country income level	No. studies	Asia-Pacific region	Pregnant women tested, no. (range†)	Women with GBS colonization, no. (range†)	Studies with GBS serotyping data, no. (%)	Studies with AMR data, no. (%)	ROB score, median (IQR)
High income	38		758,701 (79–318,740)	149,899 (17–30,176)	7 (18.4)	10 (28.9)	5 (4–6)
Australia	10	Oceania	89,828 (531–60,674)	19,253 (106–13,058)	1 (10.0)	1 (10.0)	5 (3.3–6)
Hong Kong, China	6	East Asia	435,391 (134–318,740)	89,173 (63–63,767)	0	1 (16.7)	5.5 (3.5–6.8)
Japan	9	East Asia	6,870 (79–1,957)	1,162 (17–423)	4 (44.4)	2 (22.2)	6 (4–6)
South Korea	6	East Asia	36,182 (150–33,721)	3,856 (23–3,578)	2 (33.3)	3 (50.0)	5.5 (5–6.8)
Taiwan	7	East Asia	190,430 (519–154,088)	36,455 (113–30,176)	0	3 (42.9)	5 (4–6.5)
Upper-middle income	38		210,333 (100–49,908)	22,058 (19–5,902)	10 (25.6)	13 (33.3)	5 (4–6)
China	33	East Asia	208,819 (116–49,908)	21,668 (18–5,902)	9 (26.5)	11 (32.4)	5 (4–6)
Indonesia	2	Southeast Asia	336 (159–177)	79 (26–53)	1 (50.0)	1 (50.0)	5.5 (4.3–6.8)
Malaysia	1	Southeast Asia	573	235	0	0	7
Thailand	2	Southeast Asia	605 (100–505)	76 (19–57)	0	1 (50.0)	6 (5.5–6.5)
Low and middle income	31‡		13,727 (50–3,863)	1,688 (0–310)	8 (25.8)	11 (35.5)	5 (4–6)
Bangladesh	3	South Asia	1,375 (224–1,151)	356 (46–172)	2 (66.7)	1 (33.3)	5 (5–6.5)
India	13	South Asia	4,687 (50–966)	402 (0–174)	2 (15.4)	5 (38.5)	5 (4–6)
Pakistan	5	South Asia	839 (78–261)	136 (18–48)	1 (20.0)	0	5 (4–6)
Sri Lanka	4	South Asia	825 (150–250)	142 (28–45)	1 (25.0)	3 (75.0)	5 (4.5–5.3)
Vietnam	6	Southeast Asia	6,001 (50–3,863)	652 (3−310)	2 (33.3)	2 (33.3)	6 (4.5–6.5)
Total	106‡		982,761	173,645	25 (23.5)	34 (32.1)	5 (4–6)

*Expanded data are provided in Appendix Table 2. AMR, antimicrobial drug resistance; ANC, antenatal clinic; IQR, interquartile range; ROB, risk of bias. †Range indicates the number of persons included in each study. ‡One study contributed data to both Bangladesh and India; therefore, country-specific counts exceed the total number of included studies.

Most (101 [95.3%]) studies included pregnant women who received GBS screening as part of their antenatal care visit or during labor; 3 of those studies also enrolled women experiencing preterm labor (Appendix Table 3). Data from 29 studies, predominately from Australia, Japan, Hong Kong, and Taiwan, were derived from routine antenatal screening programs. Three studies included pregnant women with preterm prelabor rupture of membranes or term premature rupture of membranes (Appendix Table 3); 2 studies included pregnant women with risk factors for GBS infection (Appendix Table 3). For GBS testing, samples were obtained from the vagina and rectum (71 [66.0%] studies), vagina only (27 [25.5%]), and vagina and cervix (2 [1.9%]); 7 (6.6%) studies did not specify the sampling site.

### GBS Colonization Prevalence

Of the 106 studies, 8 were removed from meta-analysis because of a high risk of bias score (<3) (Appendix Table 3). The 98 remaining studies in the meta-analysis included 978,150 pregnant women; 172,973 of those had GBS colonization. Overall, the pooled estimated GBS prevalence was 18.0% (95% CI 12%–23%); significant interstudy heterogeneity was observed (p = 0.001; *I^2^* = 99.8%) (Appendix Figure 1). Pooled estimated prevalences were 20.0% (95% CI 16.0%–24.0%; n = 149,539/757,142) in HICs, 11.0% (95% CI 7.0%–14.0%; n = 21,814/207,412) in UMICs, and 12.0% (95% CI 7.0%–17.0%; n = 1,579/13,462) in LMICs (Appendix Figure 1). By subregion, pooled estimated prevalences were 21.0% (95% CI 20.0%–23.0%) in Oceania, 17.0% (95% CI 12.0%–23.0%) in East Asia, 14.0% (95% CI 4.0%–24.0%) in Southeast Asia, and 13.0% (95% CI 8.0%–17.0%) in South Asia (Appendix Figure 2). We also estimated country-specific prevalences (Appendix Figure 3). We observed considerable between-study heterogeneity within countries; prevalence estimates for countries with <4 studies had wide CIs, reflecting the limited available data.

Microbiologic culture methods for GBS detection were used in 73 (74.5%) of the 98 studies; 43 (58.9%) of those 73 studies reported using enrichment or selective media. PCR alone was used in 9 (9.2%) of the 98 studies, mass spectrophotometry in 2 (2.0%) studies, and immunochromatography in 1 (1.0%) studies; GBS detection methods were not specified in 13 (13.3%) studies (Appendix Table 3). A trend toward higher GBS colonization was observed when enrichment and microbiologic culture methods were used compared with culture alone for both HICs and LMICs, but not UMICs, whereas the number of studies that used PCR was limited (Appendix Figures 4, 5).

Vaginal sampling was reported in 24 (24.5%) of the 98 studies (most were from UMICs and LMICs), whereas both vaginal and rectal samplings were reported in 65 (66.3%) studies. Two (2.0%) studies reported samples from the cervix and vagina; 7 (7.1%) did not report the sampling site. A trend toward higher GBS colonization was observed when sampling sites included both the vagina and rectum than the vagina alone for both HICs and LMICs, but not UMICs (Appendix Figures 4, 6).

### GBS Serotype Distribution

Of 98 studies included in the meta-analysis, 24 (24.5%) from 10 countries reported serotype distributions of GBS isolates (*14*–*36*) (Figure 2); 8 of those studies reported data from China. Fourteen (58.3%) of the 24 studies used multiplex PCR, and 10 (41.6%) used latex agglutination serotyping kits as their serotyping method. Six (25.0%) studies each reported serotyping data for <30 isolates, 7 (29.1%) each reported 50–100 isolates, and 11 (45.8%) each reported >100 isolates (Appendix Table 3). The 5 most reported serotypes were III (range 5.0%–100.0%), Ia (range 0%–61.0%), V (range 0.5%–32.0%), Ib (range 0.5%–22.8%), and II (range 1.5%–32.1%) (Figure 2).

**Figure 2:**
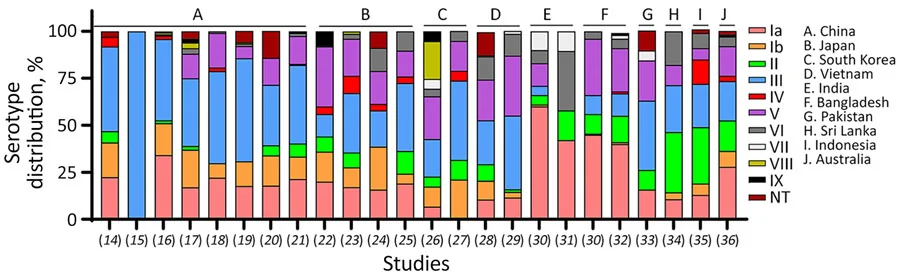
Serotype distribution of group B *Streptococcus* isolates among pregnant women, Asia-Pacific region, 2014–2025. Data are expressed as stacked bars. Colors indicate distribution of serotypes I–IX reported from 10 countries. Study reference numbers are indicated on the x-axis. NT, not typed.

### GBS AMR Patterns

A total of 30 (30.6%) of 98 studies reported GBS AMR patterns; one third of those were from China (Appendix Table 3). AMR testing varied across studies (Table 2); 12 of the 30 studies tested <50 GBS strains (Appendix Table 3). Sixteen studies reported AMR rates for both ampicillin and penicillin, the first-line antimicrobial drugs used to treat GBS infections (Table 2); an additional 11 studies reported rates only for penicillin. Overall, AMR rates were low for both penicillin and ampicillin (Table 2). Among antimicrobial drugs tested in ≥10 studies, tetracycline had the highest (median 77.7%, range 19.3%–90%) maximum AMR rate, followed by erythromycin (median 40.7%, range 5.2%–78.6%), clindamycin (median 35.7%, range 0%–77.1%), and levofloxacin (median 26.9%, range 0%–68.4%).

**Table 2:** Antimicrobial drug resistance rates for group B *Streptococcus* isolates in study of rectovaginal colonization rates and serotype distribution among pregnant women, Asia-Pacific region, 2014–2025*

Antimicrobial drugs	No. (%) studies reporting AMR†	No. isolates tested	% AMR rate, median (range)
Aminoglycosides			
Amikacin/gentamicin	2 (6.7)	74	73.2 (46.4–100)
Amphenicols			
Chloramphenicol	9 (26.5)	6,596	9.2 (2.6–52.4)
β-lactams			
Ampicillin	16 (53.3)	6,521	0 (0–2.0)
Penicillin	27 (90.0)	10,474	0 (0–2.9)
Carbapenems			
Meropenem	6 (20.0)	3,903	0 (0–2.6)
Cephalosporins			
Cefepime	3 (10.0)	3,776	2.6 (0–7.1)
Cefotaxime	15 (50.0)	5,078	0 (0–6.7)
Cefazolin	1 (3.3)	189	0
Fluoroquinolones			
Ciprofloxacin	4 (13.3)	613	8.8 (3.3–67.4)
Levofloxacin	20 (66.7)	8,919	26.9 (0–68.4)
Lincosamides			
Clindamycin	29 (96.7)	10,812	35.7 (0–77.1)
Glycopeptides			
Vancomycin	22 (73.3)	9,461	0 (0–3.3)
Teicoplanin	1 (3.3)	25	0
Macrolides			
Azithromycin	2 (6.7)	132	69.4 (51.3–87.5)
Clarithromycin	2 (6.7)	132	64.3 (50–78.6)
Erythromycin	29 (96.7)	10,348	40.7 (5.2–78.6)
Telithromycin	1 (3.3)	56	3.6
Oxazolidinone			
Linezolid	8 (26.7)	1,710	0 (0–0.8)
Streptogramin			
Quinupristin	1 (3.3)	509	0.2
Tetracyclines			
Doxycycline	2 (6.7)	74	46.2 (35.7–56.7)
Minocycline	1 (3.3)	28	56.7
Tetracycline	14 (46.7)	7,714	77.7 (19.3–90.0)

*AMR, antimicrobial drug resistance. †N = 30. Countries included in studies reporting AMR: Australia (n = 1); Bangladesh (n = 1); China (n = 10); Hong Kong, China (n = 1); India (n = 3); Indonesia (n = 1); Japan (n = 2); Taiwan (n = 3); Thailand (n = 1); South Korea (n = 3); Sri Lanka (n = 3); and Vietnam (n = 1).

### Quality of Included Studies

We determined the risk of bias scores for all 106 included studies and summarized details of the risk of bias assessment (Appendix Tables 3, 4). High risk for bias was observed in 8 (7.5%) studies, moderate risk in 53 (50.0%) studies, and low risk in 45 (42.5) studies (Appendix Table 3). Thirty (28.3%) of the 106 studies did not provide sufficient information about the study population (e.g., retrospective design or data derived from laboratory samples); 51 (48.1%) studies did not sufficiently describe the participants or settings, and 48 (45.3%) did not use appropriate methods for sample collection or GBS detection. Most (91 [85.8%]) studies used appropriate statistical methods.

## Discussion

By conducting a systematic review and meta-analysis, we estimated that the prevalence of GBS colonization among pregnant women in the Asia-Pacific region was 18.0% (95% CI 12%–23%). Approximately one third of studies reported data from China, and the remainder reported data from 12 other countries in the region. We did not find studies from the Oceania region apart from Australia, and no studies reported data for island countries within the Pacific region. We included only 8 studies from countries in Southeast Asia, and no data were reported on GBS colonization rates among pregnant women in high neonatal mortality settings (>20 deaths/1,000 live births) (*37*).

Our findings were consistent with a systematic review published in 2017, which reported a global maternal GBS colonization prevalence of 18% (95% CI 17%–19%) (*11*). Our regional estimates were also broadly consistent with those reported previously, including 21.0% in our study versus 23% for Oceania (Australia/New Zealand), 17.0% versus 11% for East Asia, 14.0% versus 14% for Southeast Asia, and 13.0% versus 13% for South Asia (*11*). However, we demonstrated higher rates of GBS colonization than has been previously used to estimate the global burden of invasive GBS disease (*2*). Data on invasive GBS disease remain limited across many countries in Southeast Asia and the Pacific regions, and accurate ascertainment of neonatal GBS disease rates is challenging because of limited access to sensitive microbiologic diagnostics, incomplete case capture, and the use of IAP, which reduces culture sensitivity. Hence, many LMICs rely on modeled estimates. The most recent estimates of invasive GBS were obtained by using maternal GBS colonization prevalences of 10.4% for Southeast Asia and 18.6% for Oceania (*2*). In our review, we found higher rates of maternal colonization, indicating the prevalence of invasive GBS in the Asia-Pacific region might have been underestimated.

Overall, we found that GBS colonization prevalence appeared slightly higher in HICs than in UMICs or LMICs within the Asia-Pacific region. Our findings remained consistent when we stratified studies according to GBS detection methods and sampling sites within each income group. Differences across settings might partly reflect variations in laboratory testing methods (e.g., using different types of enrichment broth and blood agar plates), sampling sites, and identification methods or, more broadly, population differences in antimicrobial drug use (*38*), intestinal microbiomes (*39*), and sexual behaviors (*40*). Our findings should be interpreted with caution because substantially more study participants from HICs were included than those from UMICs and LMICs. Furthermore, a large proportion of studies of UMICs and LMICs were conducted in China (UMICs) and India (LMICs); the high number of those studies might produce bias in the overall estimate. More studies are needed to better characterize maternal GBS prevalence, particularly in LMICs, which have the highest GBS disease prevalence.

Only 19 studies have been reported from 10 countries in the Asia-Pacific region in the past 10 years. The 5 most common GBS serotypes (Ia, Ib, II, III, and V) identified in our review were consistent with other reports of global GBS serotype distribution among pregnant women (*11*,*41*). Those 5 serotypes accounted for 97% of invasive isolates causing infant GBS disease globally. Serotype III was the most common cause of disease worldwide (*41*) and the major serotype causing invasive infant GBS disease in the Asia-Pacific region, although data have only been available for this region from China, Japan, and Australia (*41*). Sequence type 283 of serotype III has been linked to ongoing outbreaks of invasive adult GBS disease associated with consumption of raw fish in nonpregnant women within Asia (*42*). Although human shedding and human-to-human transmission are possible, sequence type 283 was not identified in any of the studies included in this review that reported multilocus sequence typing data (12 studies). Both trivalent and hexavalent vaccine candidates undergoing phase III clinical trials target serotypes III, Ia, and Ib, whereas hexavalent vaccines also target serotypes II, IV, and V (*43*,*44*). Those vaccines have the potential to reduce infant and maternal GBS invasive disease. Countries with the highest prevalence of invasive GBS disease and death among young infants are likely to benefit most from maternal GBS vaccination. Ongoing monitoring and serotype surveillance will be essential to ensure vaccine effectiveness and to detect potential shifts in GBS serotype distribution.

AMR is one of the top 10 global health threats identified by the World Health Organization; an estimated 5 million annual deaths have been associated with bacterial AMR (*45*). Antimicrobial drug use during labor is common (≈40% of patients) to treat or prevent urogenital infections, chorioamnionitis, and GBS colonization (*46*). Most studies included in our review demonstrated that a high proportion of GBS strains remained susceptible to the first-line antimicrobial drugs, penicillin and ampicillin, as well as second-line drugs, such as vancomycin. However, our review found high rates of resistance to clindamycin (up to 75%) and erythromycin (up to 80%), which are the drugs typically used for persons who are allergic to penicillin; we also found high rates of tetracycline resistance (up to 90%), likely resulting from treatment of other bacterial diseases (*47*). Antimicrobial drug–resistant GBS strains are an increasing global concern; rising resistance potentially compromises the prevention and treatment of invasive GBS disease and increases the potential for these strains to spread AMR to other pathogenic bacteria (*48*–*50*).

In HICs, established national GBS IAP programs (i.e., IAP given to pregnant women with risk factors for GBS disease development or for those with confirmed disease) are associated with lower rates of invasive GBS disease in neonates and GBS-related deaths in infants (*2*). In contrast, in most UMICs and LMICs that have no national IAP programs, invasive GBS disease and GBS-related deaths in infants are higher than in HICs, even with a lower overall prevalence of maternal GBS colonization (*2*). IAP programs have been associated with reduced risk for early-onset GBS disease, without evidence of increasing antimicrobial drug resistance (*6*). Our findings support the implementation of IAP programs, where feasible, in UMICs and LMICs. In settings where IAP is not feasible, maternal GBS vaccination might offer an alternative strategy for preventing neonatal GBS disease, when warranted according to disease prevalence.

The strengths of this review include a systematic approach that identified relevant literature; the incorporation of many studies enabled subgroup analyses, including geographic regions and country-income levels, providing further insight into the factors influencing GBS colonization rates. This review also included GBS serotyping and AMR rates that will inform GBS treatment and vaccine strategies. 

The first limitation of our study is that we excluded gray literature, such as theses and dissertations, committee reports, conference abstracts, and government reports, as well as non-English publications. Therefore, our findings might not fully represent the global body of evidence, especially data from non-English or nontraditional publication sources. Second, a single reviewer conducted the data extraction and risk of bias assessments, which might have introduced potential bias in the findings. Third, sampling sites and laboratory testing methods varied by country income status; thus, the independent effects of sampling site and testing method could not be distinguished from effects of country income level or geographic region. Finally, we did not perform subgroup analyses for regions within countries having the highest invasive GBS–mediated death rates in children <5 years of age because of limited data availability and heterogeneity in reporting across studies.

In conclusion, this study identified a pooled estimate of 18.0% GBS colonization among pregnant women in the Asia-Pacific region. Limited data are available for the Asia-Pacific region, particularly for countries within the Pacific region and parts of Southeast Asia, where neonatal mortality rates remain high. Current estimates of invasive GBS disease derived from maternal GBS colonization might be underestimated, especially in regions with limited data. Epidemiologic studies are urgently needed in potentially high-prevalence settings to elucidate the prevalence of maternal GBS colonization, circulating serotypes, and neonatal invasive GBS disease. Strengthening diagnostic capacity, evaluating key determinants of disease (including IAP implementation and antimicrobial drug use during pregnancy), and surveillance of circulating GBS serotypes and antimicrobial resistance patterns will be needed to guide prevention strategies and maternal GBS vaccine implementation.

Appendix Additional information for systematic review and meta-analysis of group B *Streptococcus* rectovaginal colonization rates and serotype distribution among pregnant women, Asia-Pacific region, 2014–2025.
