# Supplementary material for: Systematic Review and Meta-analysis of Group B Streptococcus Rectovaginal Colonization Rates and Serotype Distribution among Pregnant Women, Asia-Pacific Region, 2014–2025
Source: Emerg Infect Dis. 2026 Oct;32(10):1571–80. doi: 10.3201/eid3210.260687 (PMC13632831; doi:10.3201/eid3210.260687)
Supplement: Supplementary file 1 [file 26-0687-Techapp-s1.pdf]

Article DOI: https://doi.org/10.3201/eid3210.260687
EID cannot ensure accessibility for supplementary materials supplied by authors. Readers who have difficulty accessing supplementary content should contact the authors for assistance.

Systematic Review and Meta-analysis of Group B Streptococcus Rectovaginal Colonization Rates and Serotype Distribution among Pregnant Women, Asia-Pacific Region, 2014–2025

Appendix

Complete Literature Search Strategy

MEDLINE Search

1. Streptococcus agalactiae/

2. Streptococcal Infections/

3. (group-b-strep* or Streptococcus-agalactiae or Streptococcal-group-B or Streptococci-group-B or Streptococcus-group-B or Txid1311 or GBS).tw,kf.

4. One or 2 or 3

5. Carrier State/

6. (carriage or density or densities or load or biome or microbiome or coloni#ation or carrier-state or CFU or colony-forming or colony-formation or positive).tw,kf.

7. serotype*.tw,kf,hw.

8. (rectal or vaginal or vagina or rectum or perianal or peri-anal).tw,kf.

9. Five or 6 or 7 or 8

10. exp pregnant women/ or exp Pregnancy/ or exp Pregnancy Complications/ or exp Obstetrics/ or exp Breast Feeding/ or exp maternal health services/ or (parturition or ante-natal or antenatal* or pre-natal* or prenatal* or puerper* or intra-partum or intrapartum or antepartum or ante-

partum or postnatal* or post-natal* or postpartum or postpartum or peripartum or peri-partum or prepregnancy or preconception* or pre-conception* or periconception* or peri-conception* or ((preterm or prematur*) and (labor or labor)) or pregnancy or pregnancies or pregnant or perinatal or peri-natal or mother or mothers or matern* or obstetric* or childbirth or birth* or stillbirth*).tw,kf.

11. Maternal Exposure/ or ((pregnan* or gestation* or in-utero or mother* or maternal* or antenatal* or ante-natal* or prenatal* or pre-natal*) and (expose* or exposure* or transmission or transmit*)).tw,kf.

12. Prenatal Exposure Delayed Effects/ or Infectious Disease Transmission, Vertical/ or maternal-fetal exchange/

13. Ten or 11 or 12

14. (KAZAKHSTAN or KYRGYZSTAN or TAJIKISTAN or TURKMENISTAN or UZBEKISTAN or BORNEO or BRUNEI or timor-leste or cyprus or CAMBODIA or INDONESIA or LAO PEOPLE’S DEMOCRATIC REPUBLIC or MALAYSIA or MYANMAR or BURMA or PHILIPPINES or SINGAPORE or THAILAND or VIETNAM or BAHRAIN or IRAN or IRAQ or ISRAEL or JORDAN or KUWAIT or LEBANON or OMAN or QATAR or SAUDI-ARABIA or SYRIA or TURKEY or UNITED-ARAB-EMIRATES or UAE or YEMEN or AFGHANISTAN or BANGLADESH or BHUTAN or INDIA or NEPAL or PAKISTAN or SRI-LANKA or HIMALAYA* or MALDIVE* or PEOPLE’S REPUBLIC OF CHINA or JAPAN or KOREA or MONGOLIA or TAIWAN or HONG-KONG or MACAO or MACAU or OCEANIA or AUSTRALIA or NEW-ZEALAND or PACIFIC-ISLAND* or Cook-Island* or Micronesia* or Fiji or Polynesia* or Kiribati or Nauru or New-Caledonia or Niue or Palau or Papua-New-Guinea or PNG or Marshall-Island* or Samoa or Solomon-Island* or Tonga or Tuvalu or Vanuatu or azerbaijan or armenia).tw,kf,in.

15. exp asia/ or exp oceania/ or asia.tw,kf.

16. Fourteen or 15

17. Four and 9 and 13 and 16

18. limit 17 to yr = ”2014 -Current”

19. limit 18 to (case reports or comment or editorial or guideline or letter or practice guideline or preprint)

20. Eighteen not 19

Embase Search

1. Streptococcus agalactiae/

2. streptococcus infection/ or exp group b streptococcal infection/ or exp streptococcal bacteremia/

3. (group-b-strep* or Streptococcus-agalactiae or Streptococcal-group-B or Streptococci-group-B or Streptococcus-group-B or Txid1311 or GBS).tw,kf,dq.

4. One or 2 or 3

5. disease carrier/ or asymptomatic carrier/ or exp disease reservoir/

6. (carriage or density or densities or load or biome or microbiome or coloni#ation or carrier-state or CFU or colony-forming or colony-formation or positive).tw,kf,dq.

7. serotype*.tw,kf,hw,dq.

8. (rectal or vaginal or vagina or rectum or perianal or peri-anal).tw,kf,dq.

9. Five or 6 or 7 or 8

10. exp PREGNANCY/ or Pregnant woman/ or exp PREGNANCY DISORDER/ or exp OBSTETRIC PROCEDURE/ or exp BREAST FEEDING/ or exp BIRTH/ or exp CHILDBIRTH/ or maternal health service/ or maternal child health care/ or exp perinatal care/ or exp postnatal care/ or exp prenatal care/ or prepregnancy care/ or intrapartum care/ or (parturition or ante-natal or antenatal* or pre-natal* or prenatal* or puerper* or intra-partum or intrapartum or antepartum or ante-partum or postnatal* or post-natal* or postpartum or postpartum or peripartum or peri-partum or prepregnancy or preconception* or pre-conception* or periconception* or peri-conception* or ((preterm or prematur*) and (labor or labor)) or pregnancy or pregnancies or pregnant or perinatal or peri-natal or mother or mothers or matern* or obstetric* or childbirth or birth* or stillbirth*).tw,kf,dq.

11. maternal exposure/ or ((pregnan* or gestation* or in-utero or mother* or maternal* or antenatal* or ante-natal* or prenatal* or pre-natal*) and (expose* or exposure* or transmission or transmit*)).tw,kf,dq.

12. prenatal exposure/ or maternal fetal transmission/ or mother to child transmission/ or vertical transmission/

13. Ten or 11 or 12

14. (KAZAKHSTAN or KYRGYZSTAN or TAJIKISTAN or TURKMENISTAN or UZBEKISTAN or BORNEO or BRUNEI or timor-leste or cyprus or CAMBODIA or INDONESIA or LAO PEOPLE'S DEMOCRATIC REPUBLIC or MALAYSIA or MYANMAR or BURMA or PHILIPPINES or SINGAPORE or THAILAND or VIETNAM or BAHRAIN or IRAN or IRAQ or ISRAEL or JORDAN or KUWAIT or LEBANON or OMAN or QATAR or SAUDI-ARABIA or SYRIA or TURKEY or UNITED-ARAB-EMIRATES or UAE or YEMEN or AFGHANISTAN or BANGLADESH or BHUTAN or INDIA or NEPAL or PAKISTAN or SRI-LANKA or HIMALAYA* or MALDIVE* or PEOPLE'S REPUBLIC OF CHINA or JAPAN or KOREA or MONGOLIA or TAIWAN or HONG-KONG or MACAO or MACAU or OCEANIA or AUSTRALIA or NEW-ZEALAND or PACIFIC-ISLAND* or Cook-Island* or Micronesia* or Fiji or Polynesia* or Kiribati or Nauru or New-Caledonia or Niue or Palau or Papua-New-Guinea or PNG or Marshall-Island* or Samoa or Solomon-Island* or Tonga or Tuvalu or Vanuatu or azerbaijan or armenia).tw,kf,in,dq.

15. exp asia/ or exp pacific islands/ or asia.tw,kf,dq.

16. Fourteen or 15

17. Four and 9 and 13 and 16

18. limit 17 to yr = "2014 -Current"

19. limit 18 to (conference abstract or conference paper or "conference review" or editorial or letter or "preprint (unpublished, non-peer reviewed)")

20. case report/

21. Eighteen not (19 or 20)

PubMed Search

((((“group-b-strep*”[Title/Abstract] OR “Streptococcus-agalactiae”[Title/Abstract] OR “Streptococcal-group-B”[Title/Abstract] OR “Streptococci-group-B”[Title/Abstract] OR “Streptococcus-group-B”[Title/Abstract] OR “Txid1311”[Title/Abstract] OR “GBS”[Title/Abstract] OR “Streptococcus-infection*”[Title/Abstract] OR “Streptococcal-infection*”[Title/Abstract] OR “Streptococcal-bacteremia*”[Title/Abstract] OR “Streptococcus-bacteremia*”[Title/Abstract]) AND (“carriage”[Title/Abstract] OR “disease-carrier*”[Title/Abstract] OR “asymptomatic-carrier*”[Title/Abstract] OR “disease-reservoir*”[Title/Abstract] OR “density”[Title/Abstract] OR “densities”[Title/Abstract] OR “load”[Title/Abstract] OR “biome”[Title/Abstract] OR “microbiome”[Title/Abstract] OR “colonisation”[Title/Abstract] OR “colonization”[Title/Abstract] OR “carrier-state”[Title/Abstract] OR “CFU”[Title/Abstract] OR “colony-forming”[Title/Abstract] OR “colony-formation”[Title/Abstract] OR “positive”[Title/Abstract] OR “serotype*”[Title/Abstract] OR “rectal”[Title/Abstract] OR “vaginal”[Title/Abstract] OR “vagina”[Title/Abstract] OR “rectum”[Title/Abstract] OR “perianal”[Title/Abstract] OR “peri-anal”[Title/Abstract]) AND ((“parturition”[Title/Abstract] OR “ante-natal”[Title/Abstract] OR “antenatal*”[Title/Abstract] OR “pre-natal*”[Title/Abstract] OR “prenatal*”[Title/Abstract] OR “puerper*”[Title/Abstract] OR “intra-partum”[Title/Abstract] OR “intrapartum”[Title/Abstract] OR “antepartum”[Title/Abstract] OR “ante-partum”[Title/Abstract] OR “postnatal*”[Title/Abstract] OR “post-natal*”[Title/Abstract] OR “postpartum”[Title/Abstract] OR “post-partum”[Title/Abstract] OR “peripartum”[Title/Abstract] OR “peri-partum”[Title/Abstract] OR “prepregnancy”[Title/Abstract] OR “preconception*”[Title/Abstract] OR “pre-conception*”[Title/Abstract] OR “periconception*”[Title/Abstract] OR “peri-conception*”[Title/Abstract] OR “pregnancy”[Title/Abstract] OR “pregnancies”[Title/Abstract] OR “pregnant”[Title/Abstract] OR “gestation”[Title/Abstract] OR “gestational”[Title/Abstract] OR “in-utero”[Title/Abstract] OR “inutero”[Title/Abstract] OR “perinatal”[Title/Abstract] OR “peri-natal”[Title/Abstract] OR “mother”[Title/Abstract] OR “mothers”[Title/Abstract] OR “matern*”[Title/Abstract] OR “obstetric*”[Title/Abstract] OR “childbirth”[Title/Abstract] OR “birth*”[Title/Abstract] OR “stillbirth*”[Title/Abstract] OR “expose*”[Title/Abstract] OR “exposure*”[Title/Abstract] OR “transmission”[Title/Abstract] OR “transmit*”[Title/Abstract] OR “exchange*”[Title/Abstract]

OR “fetomaternal-transfusion”[Title/Abstract]) OR ((“preterm”[Title/Abstract] OR “premature*”[Title/Abstract]) AND (“labor”[Title/Abstract] OR “labour”[Title/Abstract]))) AND (“AZAKHSTAN”[Title/Abstract] OR “KYRGYZSTAN”[Title/Abstract] OR “TAJIKISTAN”[Title/Abstract] OR “TURKMENISTAN”[Title/Abstract] OR “UZBEKISTAN”[Title/Abstract] OR “BORNEO”[Title/Abstract] OR “BRUNEI”[Title/Abstract] OR “timor-leste”[Title/Abstract] OR “cyprus”[Title/Abstract] OR “CAMBODIA”[Title/Abstract] OR “INDONESIA”[Title/Abstract] OR “LAOS”[Title/Abstract] OR “MALAYSIA”[Title/Abstract] OR “MYANMAR”[Title/Abstract] OR “BURMA”[Title/Abstract] OR “PHILIPPINES”[Title/Abstract] OR “SINGAPORE”[Title/Abstract] OR “THAILAND”[Title/Abstract] OR “VIETNAM”[Title/Abstract] OR “BAHRAIN”[Title/Abstract] OR “IRAN”[Title/Abstract] OR “IRAQ”[Title/Abstract] OR “ISRAEL”[Title/Abstract] OR “JORDAN”[Title/Abstract] OR “KUWAIT”[Title/Abstract] OR “LEBANON”[Title/Abstract] OR “OMAN”[Title/Abstract] OR “QATAR”[Title/Abstract] OR “SAUDI-ARABIA”[Title/Abstract] OR “SYRIA”[Title/Abstract] OR “TURKEY”[Title/Abstract] OR “UNITED-ARAB-EMIRATES”[Title/Abstract] OR “UAE”[Title/Abstract] OR “YEMEN”[Title/Abstract] OR “AFGHANISTAN”[Title/Abstract] OR “BANGLADESH”[Title/Abstract] OR “BHUTAN”[Title/Abstract] OR “INDIA”[Title/Abstract] OR “NEPAL”[Title/Abstract] OR “PAKISTAN”[Title/Abstract] OR “SRI-LANKA”[Title/Abstract] OR “HIMALAYA*”[Title/Abstract] OR “MALDIVE*”[Title/Abstract] OR “CHINA”[Title/Abstract] OR “JAPAN”[Title/Abstract] OR “KOREA”[Title/Abstract] OR “MONGOLIA”[Title/Abstract] OR “TAIWAN”[Title/Abstract] OR “HONG-KONG”[Title/Abstract] OR “MACAO”[Title/Abstract] OR “MACAU”[Title/Abstract] OR “OCEANIA”[Title/Abstract] OR “AUSTRALIA”[Title/Abstract] OR “NEW-ZEALAND”[Title/Abstract] OR “PACIFIC-ISLAND*”[Title/Abstract] OR “Cook-Island*”[Title/Abstract] OR “Micronesia*”[Title/Abstract] OR “Fiji”[Title/Abstract] OR “Polynesia*”[Title/Abstract] OR “Kiribati”[Title/Abstract] OR “Nauru”[Title/Abstract] OR “New-Caledonia”[Title/Abstract] OR “Niue”[Title/Abstract] OR “Palau”[Title/Abstract] OR “Papua-New-Guinea”[Title/Abstract] OR “PNG”[Title/Abstract] OR “Marshall-Island*”[Title/Abstract] OR “Samoa”[Title/Abstract] OR “Solomon-Island*”[Title/Abstract] OR “Tonga”[Title/Abstract] OR “Tuvalu”[Title/Abstract] OR “Vanuatu”[Title/Abstract] OR “azerbaijan”[Title/Abstract] OR “Armenia”[Title/Abstract] OR “asia”[Title/Abstract]) AND

(NOTNLM OR publisher[sb] OR inprocess[sb] OR pubmednotmedline[sb] OR indatareview[sb] OR pubstatusaheadofprint)) AND (2014:3000/12/12[pdat])) NOT (((“group-b-strep*”[Title/Abstract] OR “Streptococcus-agalactiae”[Title/Abstract] OR “Streptococcal-group-B”[Title/Abstract] OR “Streptococci-group-B”[Title/Abstract] OR “Streptococcus-group-B”[Title/Abstract] OR “Txid1311”[Title/Abstract] OR “GBS”[Title/Abstract] OR “Streptococcus-infection*”[Title/Abstract] OR “Streptococcal-infection*”[Title/Abstract] OR “Streptococcal-bacteremia*”[Title/Abstract] OR “Streptococcus-bacteremia*”[Title/Abstract]) AND (“carriage”[Title/Abstract] OR “disease-carrier*”[Title/Abstract] OR “asymptomatic-carrier*”[Title/Abstract] OR “disease-reservoir*”[Title/Abstract] OR “density”[Title/Abstract] OR “densities”[Title/Abstract] OR “load”[Title/Abstract] OR “biome”[Title/Abstract] OR “microbiome”[Title/Abstract] OR “colonisation”[Title/Abstract] OR “colonization”[Title/Abstract] OR “carrier-state”[Title/Abstract] OR “CFU”[Title/Abstract] OR “colony-forming”[Title/Abstract] OR “colony-formation”[Title/Abstract] OR “positive”[Title/Abstract] OR “serotype*”[Title/Abstract] OR “rectal”[Title/Abstract] OR “vaginal”[Title/Abstract] OR “vagina”[Title/Abstract] OR “rectum”[Title/Abstract] OR “perianal”[Title/Abstract] OR “peri-anal”[Title/Abstract]) AND ((“parturition”[Title/Abstract] OR “ante-natal”[Title/Abstract] OR “antenatal*”[Title/Abstract] OR “pre-natal*”[Title/Abstract] OR “prenatal*”[Title/Abstract] OR “puerper*”[Title/Abstract] OR “intra-partum”[Title/Abstract] OR “intrapartum”[Title/Abstract] OR “antepartum”[Title/Abstract] OR “ante-partum”[Title/Abstract] OR “postnatal*”[Title/Abstract] OR “post-natal*”[Title/Abstract] OR “postpartum”[Title/Abstract] OR “post-partum”[Title/Abstract] OR “peripartum”[Title/Abstract] OR “peri-partum”[Title/Abstract] OR “prepregnancy”[Title/Abstract] OR “preconception*”[Title/Abstract] OR “pre-conception*”[Title/Abstract] OR “periconception*”[Title/Abstract] OR “peri-conception*”[Title/Abstract] OR “pregnancy”[Title/Abstract] OR “pregnancies”[Title/Abstract] OR “pregnant”[Title/Abstract] OR “gestation”[Title/Abstract] OR “gestational”[Title/Abstract] OR “in-utero”[Title/Abstract] OR “inutero”[Title/Abstract] OR “perinatal”[Title/Abstract] OR “peri-natal”[Title/Abstract] OR “mother”[Title/Abstract] OR “mothers”[Title/Abstract] OR “matern*”[Title/Abstract] OR “obstetric*”[Title/Abstract] OR “childbirth”[Title/Abstract] OR “birth*”[Title/Abstract] OR “stillbirth*”[Title/Abstract] OR “expose*”[Title/Abstract] OR “exposure*”[Title/Abstract] OR “transmission”[Title/Abstract] OR “transmit*”[Title/Abstract]

OR “exchange*”[Title/Abstract] OR “fetomaternal-transfusion”[Title/Abstract]) OR ((“preterm”[Title/Abstract] OR “premature*”[Title/Abstract]) AND (“labor”[Title/Abstract] OR “labour”[Title/Abstract]))) AND (“AZAKHSTAN”[Title/Abstract] OR “KYRGYZSTAN”[Title/Abstract] OR “TAJIKISTAN”[Title/Abstract] OR “TURKMENISTAN”[Title/Abstract] OR “UZBEKISTAN”[Title/Abstract] OR “BORNEO”[Title/Abstract] OR “BRUNEI”[Title/Abstract] OR “timor-leste”[Title/Abstract] OR “cyprus”[Title/Abstract] OR “CAMBODIA”[Title/Abstract] OR “INDONESIA”[Title/Abstract] OR “LAOS”[Title/Abstract] OR “MALAYSIA”[Title/Abstract] OR “MYANMAR”[Title/Abstract] OR “BURMA”[Title/Abstract] OR “PHILIPPINES”[Title/Abstract] OR “SINGAPORE”[Title/Abstract] OR “THAILAND”[Title/Abstract] OR “VIETNAM”[Title/Abstract] OR “BAHRAIN”[Title/Abstract] OR “IRAN”[Title/Abstract] OR “IRAQ”[Title/Abstract] OR “ISRAEL”[Title/Abstract] OR “JORDAN”[Title/Abstract] OR “KUWAIT”[Title/Abstract] OR “LEBANON”[Title/Abstract] OR “OMAN”[Title/Abstract] OR “QATAR”[Title/Abstract] OR “SAUDI-ARABIA”[Title/Abstract] OR “SYRIA”[Title/Abstract] OR “TURKEY”[Title/Abstract] OR “UNITED-ARAB-EMIRATES”[Title/Abstract] OR “UAE”[Title/Abstract] OR “YEMEN”[Title/Abstract] OR “AFGHANISTAN”[Title/Abstract] OR “BANGLADESH”[Title/Abstract] OR “BHUTAN”[Title/Abstract] OR “INDIA”[Title/Abstract] OR “NEPAL”[Title/Abstract] OR “PAKISTAN”[Title/Abstract] OR “SRI-LANKA”[Title/Abstract] OR “HIMALAYA*”[Title/Abstract] OR “MALDIVE*”[Title/Abstract] OR “CHINA”[Title/Abstract] OR “JAPAN”[Title/Abstract] OR “KOREA”[Title/Abstract] OR “MONGOLIA”[Title/Abstract] OR “TAIWAN”[Title/Abstract] OR “HONG-KONG”[Title/Abstract] OR “MACAO”[Title/Abstract] OR “MACAU”[Title/Abstract] OR “OCEANIA”[Title/Abstract] OR “AUSTRALIA”[Title/Abstract] OR “NEW-ZEALAND”[Title/Abstract] OR “PACIFIC-ISLAND*”[Title/Abstract] OR “Cook-Island*”[Title/Abstract] OR “Micronesia*”[Title/Abstract] OR “Fiji”[Title/Abstract] OR “Polynesia*”[Title/Abstract] OR “Kiribati”[Title/Abstract] OR “Nauru”[Title/Abstract] OR “New-Caledonia”[Title/Abstract] OR “Niue”[Title/Abstract] OR “Palau”[Title/Abstract] OR “Papua-New-Guinea”[Title/Abstract] OR “PNG”[Title/Abstract] OR “Marshall-Island*”[Title/Abstract] OR “Samoa”[Title/Abstract] OR “Solomon-Island*”[Title/Abstract] OR “Tonga”[Title/Abstract] OR “Tuvalu”[Title/Abstract] OR “Vanuatu”[Title/Abstract] OR

"azerbaijan"[Title/Abstract] OR "Armenia"[Title/Abstract] OR "asia"[Title/Abstract]) AND (NOTNLM OR publisher[sb] OR inprocess[sb] OR pubmednotmedline[sb] OR indatareview[sb] OR pubstatusaheadofprint)) AND ((booksdocs[Filter] OR casereports[Filter] OR comment[Filter] OR editorial[Filter] OR guideline[Filter] OR letter[Filter] OR practiceguideline[Filter] OR preprint[Filter]) AND (2014:3000/12/12[pdat]))))

References for All Included Studies

1. Braye K, Foureur M, de Waal K, Jones M, Putt E, Ferguson J. Group B streptococcal screening, intrapartum antibiotic prophylaxis, and neonatal early-onset infection rates in an Australian local health district: 2006–2016. PLoS One. 2019;14:e0214295. PubMed https://doi.org/10.1371/journal.pone.0214295

2. Braye K, Ferguson J, Ball J, Foureur M. Intrapartum antibiotic prophylaxis for women who are screened positive for group B streptococcal colonisation: clinical compliance with the guideline. Aust N Z J Obstet Gynaecol. 2021;61:870–5. PubMed https://doi.org/10.1111/ajo.13370

3. Chan WSW, Chua SC, Gidding HF, Ramjan D, Wong MY, Olma T, et al. Rapid identification of group B *Streptococcus* carriage by PCR to assist in the management of women with prelabour rupture of membranes in term pregnancy. Aust N Z J Obstet Gynaecol. 2014;54:138–45. PubMed https://doi.org/10.1111/ajo.12145

4. Furfaro LL, Nathan EA, Chang BJ, Payne MS. Group B *Streptococcus* prevalence, serotype distribution and colonization dynamics in Western Australian pregnant women. J Med Microbiol. 2019;68:728–40. PubMed https://doi.org/10.1099/jmm.0.000980

5. Furfaro LL, Chang BJ, Payne MS. Detection of group B *Streptococcus* during antenatal screening in Western Australia: a comparison of culture and molecular methods. J Appl Microbiol. 2019;127:598–604. PubMed https://doi.org/10.1111/jam.14331

6. McCloskey K, Vuillermin P, Carlin JB, Cheung M, Skilton MR, Tang ML, et al.; BIS Investigator Group. Perinatal microbial exposure may influence aortic intima–media thickness in early infancy. Int J Epidemiol. 2017;46:209–18. PubMed https://doi.org/10.1093/ije/dyw042

7. Mehta S, Simeunovic S, Catalano N. Relationship between group B streptococcal rectovaginal colonization and vitamin D deficiency in pregnant women. BMC Pregnancy Childbirth. 2025;25:956. PubMed https://doi.org/10.1186/s12884-025-08074-1

8. Moorhead R, Daley AJ, Lee LY, Gorelik A, Garland SM. Compliance with screening for and recommended management of maternal group B *Streptococcus* carriage in pregnancy. Aust N Z J Obstet Gynaecol. 2019;59:837–42. PubMed https://doi.org/10.1111/ajo.12977

9. Pangerl S, Sundin D, Geraghty S. Adherence to screening and management guidelines of maternal group B *Streptococcus* colonization in pregnancy. J Adv Nurs. 2022;78:3247–60. PubMed https://doi.org/10.1111/jan.15249

10. Thompson K, Yan S, Low G, Henry A. Risk factors for maternal pyrexia, infection and sepsis in four hospitals providing maternity care in New South Wales, Australia: a cohort study. Front Glob Womens Health. 2025;6:1532500. PubMed https://doi.org/10.3389/fgwh.2025.1532500

11. Chan YTV, Lau SYF, Hui SYA, Ma T, Kong CW, Kwong LT, et al.; HA-DH Liaison Committee, COC (O&G). Incidence of neonatal sepsis after universal antenatal culture-based screening of group B *Streptococcus* and intrapartum antibiotics: a multicentre retrospective cohort study. BJOG. 2023;130:24–31. PubMed https://doi.org/10.1111/1471-0528.17279

12. Fung TY, Sahota DS. How can we reduce neonatal sepsis after universal group B *Streptococcus* screening? BMC Pregnancy Childbirth. 2024;24:586. PubMed https://doi.org/10.1186/s12884-024-06791-7

13. Li YY, Kong CW, To WWK. Pathogens in preterm prelabour rupture of membranes and erythromycin for antibiotic prophylaxis: a retrospective analysis. Hong Kong Med J. 2019;25:287–94. PubMed https://doi.org/10.12809/hkmj197991

14. Ma TWL, Chan V, So CH, Hui ASY, Lee CN, Hui APW, et al. Prevention of early onset group B streptococcal disease by universal antenatal culture-based screening in all public hospitals in Hong Kong. J Matern Fetal Neonatal Med. 2018;31:881–7. PubMed https://doi.org/10.1080/14767058.2017.1300649

15. Seto MTY, Ko JKY, Cheung KW, To KKW, Hui PW, Lao TT, et al. The accuracy of self-screening of group B *Streptococcus* in pregnant women—a randomized crossover study. J Obstet Gynaecol Can. 2019;41:792–7. PubMed https://doi.org/10.1016/j.jogc.2018.08.003

16. Yeung SW, Cheung PT, Chau SL, Ip M, Lao TT, Leung TY, et al. Evaluation of an in-house real-time polymerase chain reaction method to identify group B *Streptococcus* colonization in pregnancy. J Obstet Gynaecol Res. 2015;41:1357–62. PubMed https://doi.org/10.1111/jog.12724

17. Kawaguchiya M, Urushibara N, Aung MS, Shimada S, Nakamura M, Ito M, et al. Molecular characterization and antimicrobial resistance of *Streptococcus agalactiae* isolated from pregnant

women in Japan, 2017–2021. IJID Reg. 2022;4:143–5. PubMed https://doi.org/10.1016/j.ijregi.2022.07.002

18. Morita T, Feng D, Kamio Y, Kanno I, Somaya T, Imai K, et al. Evaluation of chromID strepto B as a screening media for *Streptococcus agalactiae*. BMC Infect Dis. 2014;14:46. PubMed https://doi.org/10.1186/1471-2334-14-46

19. Nakano S, Hasegawa Y, Koide S, Hosaka Y, Ishida-Kuroki K, Kawakami S, et al. Increased recovery of *Streptococcus agalactiae* from vaginal-rectal swabs using a selective enrichment broth medium. J Obstet Gynaecol Res. 2025;51:e70084. PubMed https://doi.org/10.1111/jog.70084

20. Shibata M, Morozumi M, Maeda N, Komiyama O, Shiro H, Iwata S, et al. Relationship between intrapartum antibiotic prophylaxis and group B streptococcal colonization dynamics in Japanese mother–neonate pairs. J Infect Chemother. 2021;27:977–83. PubMed https://doi.org/10.1016/j.jiac.2021.02.006

21. Takayama Y, Matsui H, Adachi Y, Nihonyanagi S, Wada T, Mochizuki J, et al. Detection of *Streptococcus agalactiae* by immunochromatography with group B *Streptococcus*–specific surface immunogenic protein in pregnant women. J Infect Chemother. 2017;23:678–82. PubMed https://doi.org/10.1016/j.jiac.2017.07.001

22. Tanaka K, Iwashita M, Matsushima M, Wachi Y, Izawa T, Sakai K, et al. Intrapartum group B *Streptococcus* screening using real-time polymerase chain reaction in Japanese population. J Matern Fetal Neonatal Med. 2016;29:130–4. PubMed https://doi.org/10.3109/14767058.2014.989496

23. Tano S, Ueno T, Mayama M, Yamada T, Takeda T, Uno K, et al. Relationship between vaginal group B *Streptococcus* colonization in the early stage of pregnancy and preterm birth: a retrospective cohort study. BMC Pregnancy Childbirth. 2021;21:141. PubMed https://doi.org/10.1186/s12884-021-03624-9

24. Tanno D, Saito K, Tomii Y, Nakatsuka Y, Uechi K, Ohashi K, et al. A multicenter study on the utility of selective enrichment broth for detection of group B *Streptococcus* in pregnant women in Japan. Jpn J Infect Dis. 2024;77:68–74. PubMed https://doi.org/10.7883/yoken.JJID.2023.295

25. Yoshida E, Takeda J, Maruyama Y, Suga N, Takeda S, Arai H, et al. Prospective study of peripartum group B *Streptococcus* colonization in Japanese mothers and neonates. Epidemiol Infect. 2025;153:e1. PubMed https://doi.org/10.1017/S0950268824001560

26. Bae HG, Hong J, Kim YJ, Lee KR, Lee K, Choi SJ, et al. A retrospective national study on colonization rate and antimicrobial susceptibility of *Streptococcus agalactiae* in pregnant Korean women, 2018–2020. Yonsei Med J. 2022;63:717–23. PubMed https://doi.org/10.3349/ymj.2022.63.8.717

27. Choi SJ, Kang J, Uh Y. Recent epidemiological changes in group B *Streptococcus* among pregnant Korean women. Ann Lab Med. 2021;41:380–5. PubMed https://doi.org/10.3343/alm.2021.41.4.380

28. Kim DH, Min BJ, Jung EJ, Byun JM, Jeong DH, Lee KB, et al. Prevalence of group B *Streptococcus* colonization in pregnant women in a tertiary care center in Korea. Obstet Gynecol Sci. 2018;61:575–83. PubMed https://doi.org/10.5468/ogs.2018.61.5.575

29. Lee HT, Kim SY, Park PW, Ahn JY, Kim KH, Seo JY, et al. Detection and genomic analysis of genital group B *Streptococcus* in pregnant Korean women. J Obstet Gynaecol Res. 2019;45:69–77. PubMed https://doi.org/10.1111/jog.13810

30. Song KE, Hwang N, Ham JY, Cha HH, Chong GO, Lee NY. Prevalence of group B *Streptococcus* colonization in pregnant women at a University Hospital in Korea. Clin Lab. 2022;68:1711–6. PubMed https://doi.org/10.7754/Clin.Lab.2021.211126

31. Sung JH, Cha HH, Lee NY, Lee WK, Choi Y, Han HS, et al. Diagnostic accuracy of loop-mediated isothermal amplification assay for group B Streptococcus detection in recto-vaginal swab: comparison with polymerase chain reaction test and conventional culture. Diagnostics. 2022;12:1569. PubMed https://doi.org/10.3390/diagnostics12071569

32. Chang FW, Lee CI, Fan HC, Su HY, Liu YL, Chen CY. The impact of prenatal group B *Streptococcus* screening as a national health policy in Taiwan. Taiwan J Obstet Gynecol. 2017;56:648–51. PubMed https://doi.org/10.1016/j.tjog.2017.08.013

33. Cho CY, Tang YH, Chen YH, Wang SY, Yang YH, Wang TH, et al. Group B streptococcal infection in neonates and colonization in pregnant women: an epidemiological retrospective analysis. J Microbiol Immunol Infect. 2019;52:265–72. PubMed https://doi.org/10.1016/j.jmii.2017.08.004

34. Hung LC, Kung PT, Chiu TH, Su HP, Ho M, Kao HF, et al. Risk factors for neonatal early-onset group B *Streptococcus*-related diseases after the implementation of a universal screening program in Taiwan. BMC Public Health. 2018;18:438. PubMed https://doi.org/10.1186/s12889-018-5358-0

35. Lai TJ, Wang YH, Chong E, Lin YM, Huang CC, Feng KJ, et al. The impact of prenatal use of oral *Clostridium butyricum* on maternal group B *Streptococcus* colonization: a retrospective study. Taiwan J Obstet Gynecol. 2021;60:442–8. PubMed https://doi.org/10.1016/j.tjog.2021.03.009

36. Lee WT, Lai MC. High prevalence of *Streptococcus agalactiae* from vaginas of women in Taiwan and its mechanisms of macrolide and quinolone resistance. J Microbiol Immunol Infect. 2015;48:510–6. PubMed https://doi.org/10.1016/j.jmii.2014.03.002

37. Li YP, Kuok CM, Lin SY, Hsieh WS, Shyu MK. Group B *Streptococcus* antimicrobial resistance in neonates born to group B *Streptococcus*–colonized mothers: single-center survey. J Obstet Gynaecol Res. 2016;42:1471–5. PubMed https://doi.org/10.1111/jog.13082

38. Wang CH, Kung WJ, Lee CH, Lee CF, Kao CL, Chen HC, et al. High rates of colonization and antimicrobial resistance of group B *Streptococcus* highlight the need for vaccination even after implementation of guidelines for intrapartum antibiotic prophylaxis. Vaccine. 2022;40:282–7. PubMed https://doi.org/10.1016/j.vaccine.2021.11.069

39. Chen Z, Wu C, Cao X, Wen G, Guo D, Yao Z, et al. Risk factors for neonatal group B *Streptococcus* vertical transmission: a prospective cohort study of 1815 mother–baby pairs. J Perinatol. 2018;38:1309–17. PubMed https://doi.org/10.1038/s41372-018-0182-z

40. Chen J, Fu J, Du W, Liu X, Rongkavilit C, Huang X, et al. Group B streptococcal colonization in mothers and infants in western China: prevalences and risk factors. BMC Infect Dis. 2018;18:291. PubMed https://doi.org/10.1186/s12879-018-3216-4

41. Chen Z, Wen G, Cao X, Li S, Wang X, Yao Z, et al. Group B *Streptococcus* colonisation and associated risk factors among pregnant women: a hospital-based study and implications for primary care. Int J Clin Pract. 2019;73:e13276. PubMed https://doi.org/10.1111/ijcp.13276

42. Chen R, Wu L, Ma F, Chen X, Zhu Y. The accuracy and influencing factors for preference of self-sampling in group B *Streptococcus* screening: a cross-sectional study. J Matern Fetal Neonatal Med. 2022;35:5194–8. PubMed https://doi.org/10.1080/14767058.2021.1875441

43. Chen X, Cao S, Fu X, Ni Y, Huang B, Wu J, et al. The risk factors for Group B *Streptococcus* colonization during pregnancy and influences of intrapartum antibiotic prophylaxis on maternal and neonatal outcomes. BMC Pregnancy Childbirth. 2023;23:207. PubMed https://doi.org/10.1186/s12884-023-05478-9

44. Chen Y, Liu L, Liu J, Ji T, Gao Y, Yang D, et al. Serotype distribution, antimicrobial resistance, and molecular characterization of group B *Streptococcus* isolates from Chinese pregnant woman. J

Matern Fetal Neonatal Med. 2024;37:2295805. PubMed https://doi.org/10.1080/14767058.2023.2295805

45. Dai W, Zhang Y, Xu Y, Zhu M, Rong X, Zhong Q. The effect of group B *Streptococcus* on maternal and infants' prognosis in Guizhou, China. Biosci Rep. 2019;39:BSR20191575. PubMed https://doi.org/10.1042/BSR20191575

46. Du Q, Liu X, Shi T, Long J, Ma W, Huang G, et al. Clinical significance of group B *Streptococcus* testing in late pregnancy. Clin Exp Obstet Gynecol. 2016;43:703–7. PubMed https://doi.org/10.12891/ceog3123.2016

47. Gao K, Deng Q, Huang L, Chang CY, Zhong H, Xie Y, et al. Diagnostic performance of various methodologies for group B S*treptococcus* screening in pregnant woman in China. Front Cell Infect Microbiol. 2021;11:651968. PubMed https://doi.org/10.3389/fcimb.2021.651968

48. Ge Y, Pan F, Bai R, Mao Y, Ji W, Wang F, et al. Prevalence of group B *Streptococcus* colonization in pregnant women in Jiangsu, East China. BMC Infect Dis. 2021;21:492. PubMed https://doi.org/10.1186/s12879-021-06186-5

49. Ji W, Zhang L, Guo Z, Xie S, Yang W, Chen J, et al. Colonization prevalence and antibiotic susceptibility of group B *Streptococcus* in pregnant women over a 6-year period in Dongguan, China. PLoS One. 2017;12:e0183083. PubMed https://doi.org/10.1371/journal.pone.0183083

50. Ji Y, Zhao C, Ma XX, Peppelenbosch MP, Ma Z, Pan Q. Outcome of a screening program for the prevention of neonatal early-onset group B *Streptococcus* infection: a population-based cohort study in Inner Mongolia, China. J Med Microbiol. 2019;68:803–11. PubMed https://doi.org/10.1099/jmm.0.000976

51. Jiao J, Wu W, Shen F, Liu Z, Zhou H, Fan G, et al. Clinical profile and risk factors of group B streptococcal colonization in mothers from the eastern district of China. J Trop Med. 2022;2022:5236430. PubMed https://doi.org/10.1155/2022/5236430

52. Jin G, Liu L, Wang X, Fei J, Zhu Z, Jiang Z, et al. Maternal and neonatal outcomes of group B *Streptococcus* colonization: a retrospective study. BMC Infect Dis. 2025;25:94. PubMed https://doi.org/10.1186/s12879-025-10510-8

53. Lai X, Chen M, Wang J, Wang J, Lv H, Xie H, et al. CAMP-negative *Streptococcus agalactiae* strains exhibited complete or partial chromosomal deletions of the CAMP-factor encoding gene *cfb.* Microbiol Spectr. 2025;13:e0325724. PubMed https://doi.org/10.1128/spectrum.03257-24

54. Li X, Gao W, Jia Z, Yao K, Yang J, Tong J, et al. Characterization of group B *Streptococcus* recovered from pregnant women and newborns attending in a hospital in Beijing, China. Infect Drug Resist. 2023;16:2549–59. PubMed https://doi.org/10.2147/IDR.S395942

55. Li L, Wang X, Li D, Liu Z. Serum inflammatory and coagulation markers in GBS infection and adverse pregnancy outcomes. Biomark Med. 2026;20:17–25. PubMed https://doi.org/10.1080/17520363.2025.2538429

56. Liao Q, Zhang XF, Mi X, Jin F, Sun HM, Wang QX. Influence of group B *Streptococcus* and vaginal cleanliness on the vaginal microbiome of pregnant women. World J Clin Cases. 2022;10:12578–86. PubMed https://doi.org/10.12998/wjcc.v10.i34.12578

57. Liu J, Yan J, Lv J. PCR targeting 16S rRNA, *cfb*, and *hylB* for group B *Streptococcus* detection in women with 35 to 37 weeks of pregnancy. Biomark Med. 2025;19:379–84. PubMed https://doi.org/10.1080/17520363.2025.2496132

58. Liu Y, Liu W, Zhuang G, Liu W, Qiu C. Colonisation of group B *Streptococcus* and its effects on pregnancy outcomes in pregnant women in Guangzhou, China: a retrospective study. BMJ Open. 2023;13:e078759. PubMed https://doi.org/10.1136/bmjopen-2023-078759

59. Lu B, Li D, Cui Y, Sui W, Huang L, Lu X. Epidemiology of group B *Streptococcus* isolated from pregnant women in Beijing, China. Clin Microbiol Infect. 2014;20:O370–3. PubMed https://doi.org/10.1111/1469-0691.12416

60. Lu L, Chen Y, Wang Q, Gao J, Ying C. To evaluate the performance of simultaneous amplification and testing assay for group B *Streptococcus* detection: comparison with real-time PCR and ddPCR assays. Ann Clin Microbiol Antimicrob. 2024;23:65. PubMed https://doi.org/10.1186/s12941-024-00726-y

61. Ma H, Xu J, Zhang Y, Zhang R, Wu J. The current status of group B *Streptococcus* infection in the reproductive tract of late-pregnancy women and its impact on pregnancy outcomes. Arch Gynecol Obstet. 2024;310:1151–5. PubMed https://doi.org/10.1007/s00404-023-07343-8

62. Wang P, Tong JJ, Ma XH, Song FL, Fan L, Guo CM, et al. Serotypes, antibiotic susceptibilities, and multi-locus sequence type profiles of *Streptococcus agalactiae* isolates circulating in Beijing, China. PLoS One. 2015;10:e0120035. PubMed https://doi.org/10.1371/journal.pone.0120035

63. Wang X, Cao X, Li S, Ou Q, Lin D, Yao Z, et al. Phenotypic and molecular characterization of *Streptococcus agalactiae* colonized in Chinese pregnant women: predominance of ST19/III and ST17/III. Res Microbiol. 2018;169:101–7. PubMed https://doi.org/10.1016/j.resmic.2017.12.004

64. Wang YX, Zhong M, Yi H, He HF. Detection of group B streptococcus colonization in cervical and lower vaginal secretions of pregnant women. Clin Exp Obstet Gynecol. 2020;47:669–74. https://doi.org/10.31083/j.ceog.2020.05.5411

65. Wang F, Yi L, Ming F, Dong R, Wang F, Chen R, et al. Evaluation of the Xpert Xpress GBS test for rapid detection of group B *Streptococcus* in pregnant women. Microbiol Spectr. 2024;12:e0220623. PubMed https://doi.org/10.1128/spectrum.02206-23

66. Xiao X, Zheng Z, Sun H. Study on the correlation between genital tract microenvironment and GBS carrier rate of late-stage pregnant women in Dongguan. Clin Lab. 2023;69. PubMed https://doi.org/10.7754/Clin.Lab.2022.220742

67. Xie Y, Yang J, Zhao P, Jia H, Wang Q. Occurrence and detection method evaluation of group B *Streptococcus* from prenatal vaginal specimen in Northwest China. Diagn Pathol. 2016;11:8. PubMed https://doi.org/10.1186/s13000-016-0463-9

68. Yang H, Zhang S. A retrospective study comparing of group B *Streptococcus* invasiveness in pregnant women and infants. Clin Exp Obstet Gynecol. 2023;50:221. https://doi.org/10.31083/j.ceog5010221

69. Zhang H, Liu WL, Liu JL, Li MC. Identification and characteristics of antimicrobial-resistant group B *Streptococcus* isolated from maternal birth canals at a university-affiliated hospital, China. Clin Exp Obstet Gynecol. 2021;48:253–6. https://doi.org/10.31083/j.ceog.2021.02.2242

70. Zhou W, Chen X, Chen J, Zheng X, Zhang X, Chen Y, et al. Genotype distribution and high-risk factors analysis of group B *Streptococcus* in late-stage pregnant women in the Linyi Region. Int J Microbiol. 2024;2024:9910073. PubMed https://doi.org/10.1155/ijm/9910073

71. Zhu Y, Huang J, Lin XZ, Chen C. Group B *Streptococcus* colonization in late pregnancy and invasive infection in neonates in China: a population-based 3-year study. Neonatology. 2019;115:301–9. PubMed https://doi.org/10.1159/000494133

72. Safari D, Gultom SM, Tafroji W, Azzahidah A, Soesanti F, Khoeri MM, et al. Prevalence, serotype and antibiotic susceptibility of group B *Streptococcus* isolated from pregnant women in Jakarta, Indonesia. PLoS One. 2021;16:e0252328. PubMed https://doi.org/10.1371/journal.pone.0252328

73. Schwab FD, Zettler EK, Moh A, Schötzau A, Gross U, Günthert AR. Predictive factors for preterm delivery under rural conditions in post-tsunami Banda Aceh. J Perinat Med. 2016;44:511–5. PubMed https://doi.org/10.1515/jpm-2015-0004

74. Esmaon R, Lim BK, Gan F, Hamdan M, Tan PC. Sexual activity, vaginal symptoms, maternal perineal hygiene behavior, and constipation on ano-vaginal colonization of group B *Streptococcus* in near term pregnancy. BMC Pregnancy Childbirth. 2024;24:461. PubMed https://doi.org/10.1186/s12884-024-06616-7

75. Akkaneesermsaeng W, Petpichetchian C, Yingkachorn M, Sasithorn S. Prevalence and risk factors of group B *Streptococcus* colonisation in intrapartum women: a cross-sectional study. J Obstet Gynaecol. 2019;39:1093–7. PubMed https://doi.org/10.1080/01443615.2019.1587597

76. Khamching C, Boonthum A, Srisupundit K. The prevalence of group B streptococcus (GBS) colonization in pregnant women. J Med Assoc Thai. 2017;100:1162–6 [cited 2026 Mar 5]. http://www.jmatonline.com/PDF/1162-1166-PB-100-11.pdf

77. Saha SK, Ahmed ZB, Modak JK, Naziat H, Saha S, Uddin MA, et al. Group B *Streptococcus* among pregnant women and newborns in Mirzapur, Bangladesh: colonization, vertical transmission, and serotype distribution. J Clin Microbiol. 2017;55:2406–12. PubMed https://doi.org/10.1128/JCM.00380-17

78. Tarana MN, Shamsuzzaman SM. Detection of group B *Streptococcus* in vaginal swab of pregnant women by culture and PCR and their antibiotic susceptibility pattern in a tertiary care hospital of Bangladesh. Mymensingh Med J. 2018;27:567–72. PubMed

79. Kwatra G, Izu A, Cutland C, Akaba G, Ali MM, Ahmed Z, et al. Prevalence of group B *Streptococcus* colonisation in mother–newborn dyads in low-income and middle-income south Asian and African countries: a prospective, observational study. Lancet Microbe. 2024;5:100897. PubMed https://doi.org/10.1016/S2666-5247(24)00129-0

80. Ajith S, Anusree V, George B, Sherin S, Shaji P. Prevalence of group B *Streptococcus* in pregnancy and its relation to obstetric outcome in a tertiary hospital in North Kerala. Natl J Physiol Pharm Pharmacol. 2024;14:2418–22. https://doi.org/10.5455/njppp.2024.v14.i11.13

81. Faby A, Anuradha M, Rachel R, Tripathy S, Raveendran SK. Screening of group B streptococcal and *Candida* infections in antenatal women visiting a tertiary care hospital and the neonatal outcome. J Pure Appl Microbiol. 2024;18:193–9. https://doi.org/10.22207/JPAM.18.1.01

82. Geetanjali G, Shivaprakash S, Kumar S. Prevalence of group B *Streptococcus* and antimicrobial resistance in pregnant women approaching term [cited 2026 Mar 5]. https://www.ijmedph.org/Uploads/Volume15Issue1/219.%20[1682.%20IJMEDPH_Ajai]%201165-1170.pdf

83. Goel N, Wattal C, Gujral K, Dhaduk N, Mansukhani C, Garg P. Group B *Streptococcus* in Indian pregnant women: its prevalence and risk factors. Indian J Med Microbiol. 2020;38:357–61. PubMed https://doi.org/10.4103/ijmm.IJMM_20_333

84. Gogoi M, Das MK, Das JK, Barman N, Das P, Devi U, et al. Group B *Streptococcus* colonising the genital tract of pregnant women from Dibrugarh, Assam: circulating serotypes, susceptibility pattern and phylogenetic analysis. J Clin Diagn Res. 2021;15:DC13–8. https://doi.org/10.7860/JCDR/2021/47750.14824

85. Gurudas G, Arjun R, Jain N, Ranganayaki V, Sasikumar C, Mohan V, et al. Prevalence of group B *Streptococcus* in pregnant women in Kerala and relation to neonatal outcomes: a prospective cross-sectional study. J Trop Pediatr. 2022;68:fmac092. PubMed https://doi.org/10.1093/tropej/fmac092

86. Hariharan J, Venkatesan P, Shanmugam P. Incidence of vaginal colonization of group B streptococci among pregnant women attending a tertiary care hospital—Chennai, India. J Pure Appl Microbiol. 2022;16:419–23. https://doi.org/10.22207/JPAM.16.1.36

87. Konikkara KP, Baliga S, Shenoy S, Bharati B. Evaluation of culture, antigen detection and polymerase chain reaction for detection of vaginal colonization of group B *Streptococcus* (GBS) in pregnant women. J Clin Diagn Res. 2014;8:47–9. PubMed https://doi.org/10.7860/JCDR/2014/6675.4004

88. Kumar S, Kumar B, Kumari P. A microbiological study on early onset neonatal septicaemia and its correlation with the vaginal micro flora of mother in labour [cited 2026 Mar 5]. https://www.academicmed.org/Uploads/Volume5Issue6/209.%20[1625.%20JAMP_Ajay_Back]%201025-1028.pdf

89. Nubila, Vimalraj AN. Group B streptococcal colonisation in pregnant women at a tertiary care centre in central Kerala—a cross-sectional study [cited 2026 Mar 5]. https://www.academicmed.org/Uploads/Volume4Issue5/32.-77.-JAMP_Arpita-147-151.pdf

90. Warrier LM, Joy S, C RR, Bashir RA. Group B streptococcal colonization among pregnant women and neonates in a tertiary care hospital in South India. Indian J Pediatr. 2022;89:1187–94. PubMed https://doi.org/10.1007/s12098-022-04120-4

91. Santhanam S, Jose R, Sahni RD, Thomas N, Beck MM. Prevalence of group B streptococcal colonization among pregnant women and neonates in a tertiary hospital in India. J Turk Ger Gynecol Assoc. 2017;18:181–4. PubMed https://doi.org/10.4274/jtgga.2017.0032

92. Khani MN, Salat S, Ariff S, Afzal A, Ahmed S, Usman M. Risk factors associated with early onset group B streptococcal disease and newborn outcomes in a tertiary care center in Pakistan. Pak J Med Health Sci. 2023;17:576–80. https://doi.org/10.53350/pjmhs2023171576

93. Mumtaz M, Saleem S, Sattar A, Waheed A, Jahan S, Gill M. Identification of serotypes of non-typeable group b streptococci by polymerase chain reaction [cited 2026 Mar 5]. https://pjmhsonline.com/2020/jan_march/pdf/338.pdf

94. Munir SI, Waheed K, Khanum A, Iqbal R, Eusaph AZ, Hanif A. Frequency of group B streptococci in pregnant women in a tertiary care hospital. J Coll Physicians Surg Pak. 2016;26:27–30. PubMed

95. Navid S, Butt A, Aslam A, Asif S, Gul M, Ahmad S. Prevalence of group B streptococcal infection in patients with preterm premature rapture of membranes. Medical Forum Monthly. 2023;34:106–9 [cited 2026 Mar 5]. https://medicalforummonthly.com/index.php/mfm/article/view/4617

96. Wasim Z, Afsar MI, Hussain SS, Riaz T, Noor N, Shafique S. Prevalence of group B *Streptococcus* colonization in pregnant women. Pak J Med Health Sci. 2022;16:320–2. https://doi.org/10.53350/pjmhs22164320

97. Dilrukshi GN, Kottahachchi J, Dissanayake DMBT, Pathiraja RP, Karunasingha J, Sampath MKA, et al. Group B *Streptococcus* colonisation and their antimicrobial susceptibility among pregnant women attending antenatal clinics in tertiary care hospitals in the Western Province of Sri Lanka. J Obstet Gynaecol. 2021;41:1–6. PubMed https://doi.org/10.1080/01443615.2020.171631

98. Dilrukshi N, Kottahachchi J, Dissanayake T, Fernando N. Antibiotic sensitivity of group B *Streptococcus* from pregnant mothers and its association with resistance genes. Med Princ Pract. 2023;32:126–32. PubMed https://doi.org/10.1159/000530525

99. Nanayakkara D, Liyanapathirana V, Kandauda C, Gihan C, Ekanayake A, Adasooriya D. Maternal vaginal colonization with selected potential pathogens of neonatal sepsis in the era of antimicrobial resistance, a single center experience from Sri Lanka. BMC Infect Dis. 2018;18:351. PubMed https://doi.org/10.1186/s12879-018-3262-y

100. Sapugahawatte DN, Li C, Liyanapathirana V, Kandauda C, Gihan C, Zhu C, et al. Colonization of group B *Streptococcus* in pregnant women and their neonates from a Sri Lankan hospital. Pathogens. 2022;11:386. PubMed https://doi.org/10.3390/pathogens11040386

101. Ha MT, Tran-Thi-Bich H, Bui-Thi-Kim T, Nguyen-Thi ML, Vu-Tri T, Ho-Huynh TD, et al. Comparison of qPCR and chromogenic culture methods for rapid detection of group B

Streptococcus colonization in Vietnamese pregnant women. Pract Lab Med. 2024;42:e00435. PubMed https://doi.org/10.1016/j.plabm.2024.e00435

102. Hanh TQ, Van Du V, Hien PT, Chinh DD, Loi CB, Dung NM, et al. Prevalence and capsular type distribution of group B *Streptococcus* isolated from vagina of pregnant women in Nghe An province, Vietnam. Iran J Microbiol. 2020;12:11–7. PubMed

103. Nguyen VL, Dao HN, Le VTH, Nguyen AV, Ha VTT, Nguyen QTN, et al. Prevalence, risk factors, and serotypes of group B *Streptococcus* rectovaginal colonization among pregnant women: a cross-sectional study at three hospitals in Hanoi, Vietnam. Ther Adv Infect Dis. 2025;12:20499361251365028. PubMed https://doi.org/10.1177/20499361251365028

104. Nguyen ATC, Le Nguyen NT, Hoang TTA, Nguyen TT, Tran TTQ, Tran DNT, et al. Aerobic vaginitis in the third trimester and its impact on pregnancy outcomes. BMC Pregnancy Childbirth. 2022;22:432. PubMed https://doi.org/10.1186/s12884-022-04761-5

105. Tran TNL, Pham TDK, Le TG, Le TMT, Bui NN, De Tran V, et al. Antibiotic resistance of group B *Streptococcus* in pregnant women at 35–37 weeks of gestation in Southern Vietnam. Arch Balkan Medical Union. 2021;56:402–7. https://doi.org/10.31688/ABMU.2021.56.4.02

106. Van Du V, Dung PT, Toan NL, Van Mao C, Bac NT, Van Tong H, et al. Antimicrobial resistance in colonizing group B *Streptococcus* among pregnant women from a hospital in Vietnam. Sci Rep. 2021;11:20845. PubMed https://doi.org/10.1038/s41598-021-00468-3

Appendix Table 1. Joanna Briggs Institute risk of bias analysis for analytical cross-sectional studies*

Item†	Criteria	Yes	No	Unclear
1	Was the sample frame appropriate to address the target population?	Sample frame was described and approximated the target population.	Sample frame did not approximate the target population (e.g., participants do not represent general population) or was not described.	Mentioned pregnant women only without other details.
2	Were study participants recruited in an appropriate way?	Study design and recruitment strategies were described in detail.	Sampling method not reported.	Convenience sampling or retrospective analysis without sufficient detail or informed consent not reported
3	Was the sample size adequate?	Large national survey (N>500) or sample size calculation was performed and met required sample size.	Sample size not reported and small sample size <200 or required sample size not met.	Sample size calculation not performed, sample size not reported, or sample size was 200–500.
4	Were the study subjects and setting described in detail?	Participants and setting were described in sufficient detail, including inclusion and exclusion criteria.	Participant details and settings were not described	Participants and location were mentioned but not described
5	Was data analysis conducted with sufficient coverage of the identified sample?	Most (>90%) participants were included in the analysis	A large proportion (>10%) of participants were excluded in analysis	Participants included/excluded in analysis were not described.
6	Were valid methods used for the identification of the condition?	Include all the following: use flocked/dacron/rayon swab/Eswab, use selective enrichment broth, use appropriate agar media, identify by CAMP test, latex agglutination, or mass spectrometry	Molecular methods only without enrichment, latex agglutination directly from enrichment broth (without culture), or direct-from-specimen immunoassays	Testing method was not specified
7	Was the condition measured in a standard, reliable way for all participants?	Both vaginal and rectal swab collected, clinician collected, the same GBS test was used for all participants	Only vaginal or rectal samples collected, self-collected, different GBS tests were used for participants	No details were provided about sample collection.
8	Was there appropriate statistical analysis?	Include all the following: provides the information necessary to determine the numerator, denominator, prevalence estimate, and confidence interval; statistical analysis section provided, may correct for population characteristics	Does not provide the information necessary to determine the numerator and denominator	No statistical analysis section

*CAMP, Christie–Atkins–Munch-Peterson; GBS, group B *Streptococcus*.
†Total score of 6–8 items is considered low risk, 3–5 indicates moderate risk, and 0–2 reflects high risk of bias.

Appendix Table 2. Summary of included studies according to country-income status*

Country income level	No. studies	Asia-Pacific region	Pregnant women tested, no. (range†)	Women with GBS colonization, no. (range†)	Studies with GBS screening at ANC, no. (%)	Studies using rectovaginal swabs, no. (%)	Studies using standard detection,‡ no. (%)	Studies with GBS serotyping data, no. (%)	Studies with AMR data, no. (%)	ROB score, median (IQR)
High income	38		758,701 (79–318,740)	149,899 (17 – 30,176)	34 (89.5)	27 (71.1)	17 (44.7)	7 (18.4)	10 (28.9)	5 (4–6)
Australia	10	Oceania	89,828 (531–60,674)	19,253 (106 – 13,058)	7 (70.0)	7 (70.0)	4 (40.0)	1 (10.0)	1 (10.0)	5 (3.3–6)
Hong Kong Special Administrative Region, China	6	East Asia	435,391 (134–318,740)	89,173 (63 – 63,767)	5 (83.3)	5 (83.3)	4 (66.7)	0	1 (16.7)	5.5 (3.5–6.8)
Japan	9	East Asia	6870 (79–1,957)	1162 (17 – 423)	9 (100)	6 (66.7)	4 (44.4)	4 (44.4)	2 (22.2)	6 (4–6)
South Korea	6	East Asia	36,182 (150–33,721)	3,856 (23 – 3,578)	6 (100)	5 (83.3)	4 (66.7)	2 (33.3)	3 (50.0)	5.5 (5–6.8)
Taiwan	7	East Asia	190,430 (519–154,088)	36,455 (113 – 30,176)	7 (100)	4 (57.1)	1 (14.3)	0	3 (42.9)	5 (4–6.5)
Upper-middle income	38		210,333 (100–49,908)	22,058 (19 – 5,902)	33 (86.8)	26 (69.2)	14 (36.8)	10 (25.6)	13 (33.3)	5 (4–6)
China	33	East Asia	208,819 (116–49,908)	21,668 (18 – 5,902)	29 (87.9)	23 (69.7)	11 (33.3)	9 (26.5)	11 (32.4)	5 (4–6)
Indonesia	2	Southeast Asia	336 (159–177)	79 (26 – 53)	2 (100)	0	1 (50.0)	1 (50.0)	1 (50.0)	5.5 (4.3–6.8)
Malaysia	1	Southeast Asia	573	235	1 (100)	1 (100)	0	0	0	7
Thailand	2	Southeast Asia	605 (100–505)	76 (19 – 57)	1 (50.0)	2 (100)	2 (100)	0	1 (50.0)	6 (5.5–6.5)
Low and middle income	31§		13,727 (50–3,863)	1,688 (0 - 310)	24 (77.4)	18 (58.1)	16 (51.6)	8 (25.8)	11 (35.5)	5 (4–6)
Bangladesh	3	South Asia	1,375 (224–1,151)	356 (46 – 172)	2 (66.7)	2 (66.7)	2 (66.7)	2 (66.7)	1 (33.3)	5 (5–6.5)
India	13	South Asia	4,687 (50–966)	402 (0 – 174)	12 (84.6)	8 (61.5)	10 (76.9)	2 (15.4)	5 (38.5)	5 (4–6)
Pakistan	5	South Asia	839 (78–261)	136 (18 – 48)	2 (40.0)	2 (40.0)	0	1 (20.0)	0	5 (4–6)
Sri Lanka	4	South Asia	825 (150–250)	142 (28 – 45)	2 (50.0)	2 (50.0)	2 (50.0)	1 (25.0)	3 (75.0)	5 (4.5–5.3)
Vietnam	6	Southeast Asia	6,001 (50–3,863)	652 (3 −310)	6 (100)	4 (66.7)	2 (33.3)	2 (33.3)	2 (33.3)	6 (4.5–6.5)
Total	106§		982,761	173,645	91 (85.8)	71 (67.0)	47 (44.3)	25 (23.5)	34 (32.1)	5 (4–6)

*AMR, antimicrobial drug resistance; ANC, antenatal clinic; IQR, interquartile range; ROB, risk of bias.
†Range indicates the number of persons included in each study.
‡Standard detection method was selective enrichment broth followed by microbiologic culture.
§One study contributed data to both Bangladesh and India; therefore, country-specific counts exceed the total number of included studies.

Appendix Table 3. Complete characteristics of included studies and outcome measures by country income status*

Reference	Country, City/State/ Region†	Design	Study population	No. pregnant women tested	Gestational age at time of sampling, wk	Sampling site	GBS detection method‡	Prevalence of women with GBS colonization, no. (%)	Serotyping methods; serotype prevalence (%)	AMR testing methods; AMR prevalence (%)	ROB score
High-income countries											
Braye 2019 (*1*)	Australia, Hunter New England	Retrospective cohort	Women (age range not specified), postpartum	60,674	≥35	NS	NS	13,058 (21.5)	NS	NS	3
Braye 2021 (*2*)	Australia, New South Wales	Retrospective analysis	Women (age range not specified) attending ANC	2,322	≥35	NS	NS	526 (22.7)	NS	NS	4
Chan 2014 (*3*)	Australia, Sydney	Prospective cohort	Women (age, 15–46 y) with TPROM	574	≥37	Lower vagina and anus	Enrichment (THB with nalidixic acid), culture (horse blood agar, slide agglutination)	130 (22.6)	NS	NS	5
Furfaro 2019 (*4*)	Australia, Subiaco	Prospective cohort	Women (age, 16–50 y) attending ANC	567	≥33	Vagina and rectum	Enrichment (Strep B carrot broth), culture (CHROMagar)	140 (24.7)	Multiplex PCR (N = 140); serotype Ia (27.9), III (20.9), II (16.3), V (15.8), Ib (8.4), VI (5.1), IV (2.8), NT (1.9), VIII (0.5), IX (0.5)	NS	3
Furfaro 2019 (*5*)	Australia, Subiaco	Retrospective analysis	Women (age, ≥16 y) attending ANC	531	33–39	Vagina and rectum	Enrichment (Strep B carrot broth), culture (CHROMagar)	106 (20.0)	NS	NS	6
McCloskey 2017 (*6*)	Australia, Geelong	Prospective cohort	Women (age range not specified) attending ANC	757	35–37	Lower vagina and rectum	NS	156 (20.6)	NS	NS	7
Mehta 2025 (*7*)	Australia, Perth	Retrospective analysis	Women (age range not specified) attending ANC	795	35–37	Low vagina and rectum	Enrichment (THB with colistin/nalidixic acid broth), culture (neomycin sulfate agar)	139 (17.5)	NS	NS	5

Reference	Country, City/State/ Region†	Design	Study population	No. pregnant women tested	Gestational age at time of sampling, wk	Sampling site	GBS detection method‡	Prevalence of women with GBS colonization, no. (%)	Serotyping methods; serotype prevalence (%)	AMR testing methods; AMR prevalence (%)	ROB score
Moorhead 2019 (*8*)	Australia, Melbourne	Retrospective analysis	Women (age range not specified) attending ANC and postpartum	558	≥35	Vagina and rectum	NS	109 (19.5)	NS	NS	6
Pangerl 2022 (*9*)	Australia, Western Australia	Retrospective analysis	Women (age, 13–53 y) attending ANC and postpartum	17,999	NA	Vagina and rectum	Culture (not described)	3,907 (21.7)	NS	NS	6
Thompson 2025 (*10*)	Australia, New South Wales	Retrospective analysis	Women (age, ≥18 y) undergoing labor	5,051	≥20	NS	NS	982 (19.4)	NS	NS method (N = 393); PEN (0), AMP (0), ERY (33.4), CLI (30.9)	3
Chan 2023 (*11*)	Hong Kong Special Administrative Region, People's Republic of China	Retrospective cohort	Women (age range not specified) attending ANC	318,740	35–37	Lower vagina and rectum	Enrichment broth, culture (NS)	63,767 (20.0)	NS	NS	7
Fung 2024 (*12*)	Hong Kong Special Administrative Region, People's Republic of China	Retrospective analysis	Women (age range not specified) attending ANC and postpartum	1,553	NS	NS	NS	368 (23.7)	NS	NS	5
Li 2019 (*13*)	Hong Kong Special Administrative Region, People's Republic of China	Retrospective analysis	Women (age range not specified) with PPROM diagnosis	553	≥24	Lower vagina and rectum	NS	81 (14.6)	NS	Culture (N = 45–77); PEN (0), ERY (42.2)	3
Ma 2018 (*14*)	Hong Kong Special Administrative Region, People's Republic of China	Retrospective cohort	Women (age range not specified) attending ANC	113,989	35–37	Lower vagina and rectum	Enrichment broth (NS), culture (NS)	24,833 (21.8)	NS	NS	6

Reference	Country, City/State/ Region†	Design	Study population	No. pregnant women tested	Gestational age at time of sampling, wk	Sampling site	GBS detection method‡	Prevalence of women with GBS colonization, no. (%)	Serotyping methods; serotype prevalence (%)	AMR testing methods; AMR prevalence (%)	ROB score
Seto 2019 (*15*)	Hong Kong Special Administrative Region, People's Republic of China	Prospective cohort	Women (age range not described) attending ANC	422	35–37	Lower vagina and rectum	Enrichment (THB with nalidixic acid), culture (chromogenic agar)	83 (19.7)	NS	NS	3
Yeung 2015 (*16*)	Hong Kong Special Administrative Region, People's Republic of China	Cross-sectional	Women (age range not specified) attending ANC or admitted to antenatal ward	134	35–37	Vagina and rectum	Enrichment (THB with nalidixic acid), culture (Columbia blood agar)	41 (30.6)	NS	NS	7
Kawaguchiya 2022 (*17*)	Japan, Hokkaido	Retrospective analysis	Women (age range not specified) attending ANC	1,090	NS	NS	MALDI-TOF MS	76 (7.0)	Multiplex PCR (N = 76); serotype III (31.6), V (19.7), Ia (17.1), Ib (10.5).	Microdilution method (N = 76); β-lactams (0), resistant: LVX (15.8)	6
Morita 2014 (*18*)	Japan, Hiroo	Cross-sectional	Women (age range not specified) attending ANC	1,425	35–37	Vagina and anus	Enrichment (THB with colistin/nalidixic acid broth), culture (chromID Strepto B and blood agar)	319 (22.4)	NS	NS	2
Nakano 2024 (*19*)	Japan, Nagasaki	Prospective	Women (age range not specified) attending ANC	133	35–37	Vagina and rectum	Enrichment (THB with colistin/nalidixic acid broth), culture (chromID Strepto B and blood agar followed by MALDI-TOF MS)	25 (18.8)	Strep B latex kit (N = 25); serotype Ia (20), Ib (16), II (8), III (12), IV (4), V (32), IX (8)	Microdilution method (N = 25); PEN (0), AMP (0), CTX (0), MEM (0), TEC (0), VAN (0). ERY (48), CLI (36), LFX (24)	4
Shibata 2021 (*20*)	Japan, Tokyo	Prospective cohort	Women (age range not described) attending	251	33–37	Vagina and rectum	Culture (sheep blood agar) or PCR	57 (22.7)	Multiplex PCR (N = 57); serotype Ib (22.8), III (19.3), V	NS	8

Reference	Country, City/State/ Region†	Design	Study population	No. pregnant women tested	Gestational age at time of sampling, wk	Sampling site	GBS detection method‡	Prevalence of women with GBS colonization, no. (%)	Serotyping methods; serotype prevalence (%)	AMR testing methods; AMR prevalence (%)	ROB score
			ANC and postpartum						(17.5), Ia (15.8), VI (12.3), ND (8.8), IV (2.5)		
Takayama 2017 (*21*)	Japan, Kanagawa	Prospective cohort	Women (age, 16–44 y) attending ANC	377	35–37	Cervix and vagina	Enrichment (GBS medium), culture (chromID Strepto B medium and MALDI Biotyper)	58 (15.4)	Multiplex PCR (N = 58); serotype Ia (19.0), Ib (5.2), II (12.1), III (36.2), IV (3.4), V (13.8), VI (10.3)	NS	6
Tanaka 2016 (*22*)	Japan, Mitaka	Prospective cohort	Women (age 25–45 y) attending ANC	79	35–37	Vagina and rectum	Culture (TSA II 5% sheep blood agar M and latex agglutination)	17 (21.5)	NS	NS	4
Tano 2021 (*23*)	Japan, Toyota	Retrospective cohort	Women (age range not specified) attending routine ANC	1,033	33–37	Vagina	Culture (sheep blood agar, Gram stain, latex agglutination)	98 (9.5)	NS	NS	6
Tanno 2024 (*24*)	Japan, Fukushima, Osaka, Okinawa	Prospective	Women (age range not specified) attending ANC	1,957	35–37	Vagina and rectum	Enrichment (LIM broth), culture (sheep blood agar and latex agglutination)	423 (21.6)	NS	NS	5
Yoshida 2025 (*25*)	Japan, Tokyo	Prospective	Women (age range not specified) attending ANC and postpartum	525	>34	Vagina and rectum	Culture (CHROM-B agar plate and sheep blood agar, latex agglutination)	89 (17.0)	NS	NS	6
Bae 2022 (*26*)	South Korea	Retrospective analysis	Women (age, 25–38 y) attending ANC	33,721	35–37	Vagina	Culture (5% blood agar plate, Microflex MALDI Biotyper)	3,578 (10.6)	NS	VITEK2 automated system (N = 3,512); AMP, PEN, VAN, CTX, CRO (all 0), resistance: FEP, MEM, CHL (2.6–2.7), LVX (18.2–19.6), CLI (33.4–	8

Reference	Country, City/State/ Region†	Design	Study population	No. pregnant women tested	Gestational age at time of sampling, wk	Sampling site	GBS detection method‡	Prevalence of women with GBS colonization, no. (%)	Serotyping methods; serotype prevalence (%)	AMR testing methods; AMR prevalence (%)	ROB score
										35.7), ERY (35.6–36.8), and TET (50.0–53.3)	
Choi 2021 (*27*)	South Korea, Wonju	Prospective cohort	Women (age, 17–45 y) attending ANC or had preterm labor	379	≥35 or preterm	Vagina and ano-rectum	Enrichment (Strep B carrot or THB selective medium), culture (sheep blood agar or Granada agar medium, followed by CAMP test and latex agglutination)	75 (19.8)	Strep B latex kit (N = 75); serotype V (22.7), VIII (20.0), III (20.0). Total prevalence of serotypes VI-IX was 34.7%.	MICroSTREP and microdilution method (N = 75); AMP, PEN, CTX, CRO, FEP, MEM, VAN (all 0), resistant: LVX (20.0), CLI (16.0), ERY (28.0), TET (42.7), CHL (4.0)	5
Kim 2018 (*28*)	South Korea, Busan Paik	Cross-sectional	Women (age, >20 y) attending ANC or at risk for preterm labor	1,014	35–37	Lower vagina and anus	Culture (CHROM-B agar medium and VITEK2)	117 (11.6)	NS	NS	3
Lee 2019 (*29*)	South Korea, Seongnam	Prospective cohort	Women (age, 23–40 y) attending ANC	150	16–40	Vagina and rectum	Enrichment (THB with colistin/nalidixic acid broth), culture (chromID Strepto B and blood agar, followed by morphology assessment with/without latex agglutination and VITEK2)	26 (17.3)	Multiplex PCR (N = 19); serotype III (42.1), Ib (21.1), V (15.8), II (10.5), IV (5.3), VI (5.3)	VITECH 2 method (N = 21); PEN, VAN (each 0), resistance: TET (47.6), CLI (33.3), ERY (19.1), LVX (19.1)	7
Song 2022 (*30*)	South Korea, Daegu	Prospective cohort	Women (age, 16–44 y) attending ANC	391	35–37	Vagina and rectum	Enrichment (LIM broth), culture (5% sheep blood agar, biochemical test, VITEK2 and MALDI-TOF)	37 (9.5)	NS	NS	6

Reference	Country, City/State/ Region†	Design	Study population	No. pregnant women tested	Gestational age at time of sampling, wk	Sampling site	GBS detection method‡	Prevalence of women with GBS colonization, no. (%)	Serotyping methods; serotype prevalence (%)	AMR testing methods; AMR prevalence (%)	ROB score
Sung 2022 (*31*)	South Korea, Daegu	Cross-sectional	Women (age range not specified) attending ANC orin labor	527	≥35	Lower vagina, perineal region, and rectum	Enrichment (LIM broth), culture (5% sheep blood agar, biochemical test, VITEK2 and MALDI-TOF)	23 (4.4)	NS	NS	5
Chang 2017 (*32*)	Taiwan	Retrospective analysis	Women (age, 20–49 y) attending ANC	9,581	Not available	vagina and rectum	NS	789 (8.2)	NS	NS	4
Cho 2019 (*33*)	Taiwan, Taipei	Retrospective analysis	Women (age not specified) attending ANC	6,769	35–37	NS	NS	1,532 (22.6)	NS	NS	7
Hung 2018 (*34*)	Taiwan	Retrospective analysis	Women (age range not specified) attending ANC and postpartum	154,088	35–37	NS	NS	30,176 (19.6)	NS	NS	3
Lai 2021 (*35*)	Taiwan	Retrospective analysis	Women (age range not specified) attending ANC	1,277	36	Vagina and rectum	Culture (Strep B carrot broth and CAMP test)	208 (16.3)	NS	NS	5
Lee 2015 (*36*)	Taiwan, Tainan	Cross-sectional	Women (age range not specified) attending ANC	519	35–37	Vagina	Phoenix 100 automated bacterial identification system and MALDI-TOF	113 (21.8)	NS	Phoenix 100 system and MIC method (N = 113); resistant: FEP (7.1), CHL (13.3), CLI (35.4), ERY (43.4), LVX (33.5), MEM (0.9), PEN (0), TET (85), VAN (0).	4
Li 2016 (*37*)	Taiwan	Retrospective analysis	Women (age range not specified) attending ANC	12,200	35–37	Lower vagina and rectum	Nonenriched method and culture (NS)	2,529 (20.7)	NS	Mueller Hinton agar and disk diffusion method (N =	6

Reference	Country, City/State/ Region†	Design	Study population	No. pregnant women tested	Gestational age at time of sampling, wk	Sampling site	GBS detection method‡	Prevalence of women with GBS colonization, no. (%)	Serotyping methods; serotype prevalence (%)	AMR testing methods; AMR prevalence (%)	ROB score
										2,529); resistant: TET (87.5), CLI (49.5), ERY (49.5), CHL (9.2), LVX (3.1), PEN (0.8), VAN (0.3).	
Wang 2022 (*38*)	Taiwan, Donggang	Retrospective analysis	Women (age, <20 to ≥35 y) attending ANC	5,996	35–37	Vagina and rectum	Enrichment (LIM broth) and culture (GBS chromogenic agar medium and blood agar plate, VITEK 2)	1,108 (18.5)	NS	Disk diffusion method (N = 1108); All susceptible to PEN, AMP, VAN. resistant: CLI (42.3), ERY (40.3).	7
Upper-middle–income countries											
Chen 2018 (*39*)	People's Republic of China, Shenzhen	Cross-sectional	Women undergoing labor	1,815	≥28	Lower vagina	Enrichment (THB with colistin/nalidixic acid broth) and culture (sheep blood agar, morphological and biochemical test)	92 (5.1)	NS	NS	7
Chen 2018 (*40*)	People's Republic of China, Liuzhou	Prospective cohort	Women (age not specified) attending ANC	3,439	35–37 or at labor	Vagina and rectum	Enrichment (THB with nalidixic acid broth) and culture (sheep blood agar, morphological test, biochemical test and VITEK2)	210 (6.1)	NS	NS	4
Chen 2019 (*41*)	People's Republic of China, Shenzhen	Cross-sectional	Women (age, 17–45 y) attending ANC	2,121	≥28	Lower vagina	Enrichment (THB with colistin/nalidixic acid broth) and culture (sheep blood agar, morphological and biochemical test)	104 (4.9)	NS	NS	8

Reference	Country, City/State/ Region†	Design	Study population	No. pregnant women tested	Gestational age at time of sampling, wk	Sampling site	GBS detection method‡	Prevalence of women with GBS colonization, no. (%)	Serotyping methods; serotype prevalence (%)	AMR testing methods; AMR prevalence (%)	ROB score
Chen 2022 (*42*)	People's Republic of China, Shenzhen	Cross-sectional	Women (age, 20–43 y) attending ANC	520	35–40	Vagina and rectum	NS	18 (3.5)	NS	NS	6
Chen 2023 (*43*)	People's Republic of China, Xiamen	Retrospective analysis	Women (age, 16–56 y) attending ANC and postpartum	43,822	28–37	Vagina and rectum	Enrichment (GBS enrichment media) and culture (GBS carrot agar, CAMP test, or MALDI-TOF MS)	5,902 (13.5)	NS	NS	7
Chen 2024 (*44*)	People's Republic of China, Beijing	Cross-sectional	Women (age not specified) attending ANC	6,012	35–37	Lower vagina and rectum	Culture (Colombia agar with sheep blood and biochemical test or VITEK2)	311 (5.2)	PCR (N = 311); serotype III (43.1), Ia (34.1), Ib (17.0), followed by IV (1.9), II (1.6), VIII (1.0), V (0.6), NT (0.6).	Kirby-Bauer and D test method (N = 311); resistant: TET (81.0), ERY (76.2), CLI (64.0), LVX (50.80)	6
Dai 2019 (*45*)	People's Republic of China, Guizhou	Cross-sectional	Women (age, 20–46 y) attending ANC	380	35–37	Vagina and rectum	Enrichment followed by PCR	48 (12.6)	NS	NS	3
Du 2016 (*46*)	People's Republic of China, Chengdu	Cross-sectional	Women (age, 21–45 y) attending ANC	1,540	34–37	Vagina and rectum	PCR	86 (5.6)^	NS	NS	8
Gao 2021 (*47*)	People's Republic of China, Guangzhou	Cross-sectional	Women (age range not specified) attending ANC	1,281	35–37	Lower vagina and rectum	Enrichment (Lim broth) and culture (sheep blood agar, biochemical test and VITEK2)	130 (10.1)	NS	NS	6
Ge 2021 (*48*)	People's Republic of China, Jiangsu	Retrospective analysis	Women (age, 16–47 y) attending ANC	7,162	34–37	Vagina and rectum	Culture (THB broth followed by sheep blood agar)	249 (3.5)	NS	VITEK 2 and disk diffusion method (N = 249); All susceptible to PEN, LNZ, VAN. resistant: ERY (77.5), CLI (68.3), LVX (52.2)	6

Reference	Country, City/State/ Region†	Design	Study population	No. pregnant women tested	Gestational age at time of sampling, wk	Sampling site	GBS detection method‡	Prevalence of women with GBS colonization, no. (%)	Serotyping methods; serotype prevalence (%)	AMR testing methods; AMR prevalence (%)	ROB score
Ji 2017 (*49*)	People's Republic of China, Dongguan	Retrospective analysis	Women (age, 16–47 y) attending ANC	7,726	35–37	Vagina and rectum	Enrichment (selective enrichment broth containing gentamicin) and culture (Columbia blood agar, morphology assessment, CAMP test, and VITEK2)	636 (8.2)	Strep B latex kit (N = 153 isolates serotyped) serotype III (54.9), Ia (17.7), Ib (13.1), V (6.5), VI (1.3), VIII (0.7)	Kirby Bauer disk diffusion (N = 636); All isolates susceptible to PEN, CTX, LNZ, VAN. resistant: ERY (64.9), CLI (52.4), LVX (25.9)	6
Ji 2019 (*50*)	People's Republic of China, Inner Mongolia	Retrospective cohort	Women (age, 16–45 y) attending ANC and postpartum	9,770	35–37 and those in labor at <35	Lower vagina and ano-rectum	PCR	266 (2.7)	NS	NS	4
Jiao 2022 (*51*)	People's Republic of China, Jinan	Cross-sectional	Women (age, 18–45 y) attending ANC	2,761	>34	Vagina and anus	Culture (chromogenic agar plate without preenrichment)	185 (6.7)	Multiplex PCR (N = 172 swabs); serotypes Ia (61.0%), III (29.7%), VI (4.6%), II (3.5%), VII (0.6%) and NT (0.6%).	Drug sensitive culture plates (BD Phoenix-100 system). (N = 172); All susceptible to VAN and LNZ. resistant: ERY (80.2), CLI (75.0), LVX (65.1), TET (57.6), multidrug resistance (73.8)	2
Jin 2025 (*52*)	People's Republic of China, Jiangsu	Retrospective analysis	Women (age range not specified) attending ANC	5,183	35–37	Lower vagina and rectum	PCR	551 (10.6)	NS	NS	5
Lai 2025 (*53*)	People's Republic of China, Fujian	Retrospective analysis	Women (age range not specified) attending ANC	5,794	35–37	Vagina	Culture (chromogenic agar plate, sheep blood agar followed by CAMP test,	526 (9.1)	Capsular and sequence typing (N = 19); serotype III (100).	VITEK-2 system (N = 509); PEN (2.9), AMP (0.4), LNZ (0.8), QUI	3

Reference	Country, City/State/ Region†	Design	Study population	No. pregnant women tested	Gestational age at time of sampling, wk	Sampling site	GBS detection method‡	Prevalence of women with GBS colonization, no. (%)	Serotyping methods; serotype prevalence (%)	AMR testing methods; AMR prevalence (%)	ROB score
							VITEK2, and MALDI-TOF, DNA sequencing, and PCR)			(0.2), CIP (67.4), LVX (68.4), CLI (29.6), TET (19.3)	
Li 2023 (*54*)	People's Republic of China, Beijing	Cross-sectional	Women (age range not specified) attending ANC	1,470	35–37	Vagina and rectum	Enrichment (LIM broth) and culture (GBS differential agar and StrepB CHROM agar, morphology assessment and CAMP test)	102 (6.9)	Streptococcal grouping kit (N = 102); serotype III (36.3), Ib (20.6), Ia (16.7), V (12.7), VI (2.9), VIII (2.9%), II (2.0), IX (2.0).	Dilution method and microbial ID/AST System DL-96II (N = 102); All susceptible to AMP, PEN, CTX, VAN, LNZ, MEM. resistant: ERY (70.6), CLI (49.0), TET (78.4), LVX (45.1)	4
Li 2025 (*55*)	People's Republic of China, Jiangxi	Retrospective analysis	Women (age, 21–43 y) undergoing labor	2,200	≥35	Vagina and rectum	Culture (blood agar plate followed by VITEK2)	593 (27.0)	Latex agglutination (N = 593); serotype Ia (22.4), Ib (18.4), II (6.1), III (45.0), V (5.1), others (3.0)	NS	7
Liao 2022 (*56*)	People's Republic of China, Beijing	Cross-sectional	Women (age range not specified) attending hospital	160	NS	Vagina	NS	59 (36.9)	NS	NS	2
Liu 2025 (*57*)	People's Republic of China, Zhuhai	Prospective cohort	Women (age, 20–44 y) attending ANC	116	35–37	Vagina	Culture (blood agar plate followed by VITEK2)	21 (18.1)	NS	NS	3
Liu 2023 (*58*)	People's Republic of China, Guangzhou	Retrospective cohort	Women (age, 15–51 y) attending ANC	15,040	35–37	Vagina and rectum	Immunochroma-tography followed by culture (blood agar plate and	1,445 (9.6)	NS	NS	5

Reference	Country, City/State/ Region†	Design	Study population	No. pregnant women tested	Gestational age at time of sampling, wk	Sampling site	GBS detection method‡	Prevalence of women with GBS colonization, no. (%)	Serotyping methods; serotype prevalence (%)	AMR testing methods; AMR prevalence (%)	ROB score
							chromogenic agar plate, morphology assessment, and biochemical test)				
Lu 2014 (*59*)	People's Republic of China, Beijing	Cross-sectional	Women (age, 19–42 y) attending ANC	2,850	35–37	Vagina and rectum	Culture (blood agar plates, Gram stain, CAMP test, and VITEK2)	201 (7.1)	PCR (N = 201); serotype III (41.8), Ia (21.4), V (14.9), Ib (11.9), II (7.0), VI (1.5), IV (0.5), VI (0.5), VIII (0.5), NT (N = 1, 0.5%), VII and IX (0%).	Disk diffusion method and Phoenix 100 System (N = 201); All susceptible to PEN, AMP, VAN. resistant: CLI (55.7), ERY (66.2)	5
Lu 2024 (*60*)	People's Republic of China, Shanghai	Prospective cohort	Women (age range not specified) attending ANC	1,474	35–37	Vagina and anus	PCR	169 (11.5)	NS	NS	3
Ma 2024 (*61*)	People's Republic of China, Shanghai	Cross-sectional	Women (age, 20–43 y) attending ANC	12,132	≥32	Vagina	Enrichment (GBS enrichment media), culture (sheep blood agar and GBS color plate), and VITEK2	791 (6.5)	NS	NS	4
Wang 2015 (*62*)	People's Republic of China, Beijing	Cross-sectional	Women (age range not specified) attending ANC	863	35–37	Lower vagina and rectum	Culture (TSA with sheep blood, biochemical test and latex agglutination)	56 (6.5)	StrepB latex kit (N = 56); serotype III (32.1), Ia (17.9), Ib (16.1), V (14.3), II (5.4), NT (14.3)	E-test method and disk diffusion method (N = 56); resistant: ERY (78.6), AZI (87.5), CLA (78.6), TEL (3.6), TET (83.9), CLI (64.3), LVX (35.7)	4
Wang 2018 (*63*)	People's Republic of	Cross-sectional	Women (age, 17–45 y) attending	2,121	≥28	Lower vagina	Enrichment (THB with colistin/nalidixic	104 (4.9)	Multiplex PCR (N = 104); serotype III	Disk diffusion method and D-test	5

Reference	Country, City/State/ Region†	Design	Study population	No. pregnant women tested	Gestational age at time of sampling, wk	Sampling site	GBS detection method‡	Prevalence of women with GBS colonization, no. (%)	Serotyping methods; serotype prevalence (%)	AMR testing methods; AMR prevalence (%)	ROB score
	China, Shenzhen		hospitals for ANC				acid broth) and culture (sheep blood agar, morphological and biochemical tests)		(49.0), Ia (22.1), V (18.3), Ib (7.7), IV (1.9), VI (1.0)	method (N = 104); All susceptible to PEN, VAN, CTX, LNZ. resistant: TET (76.9), ERY (72.1), CLI (66.4), LVX (27.9), CHL (8.7).	
Wang 2020 (*64*)	People's Republic of China, Shenzhen	Prospective cohort	Women (age, ≈27 y) attending outpatient clinic	1,852	37	Cervix and lower vagina	Colloidal gold immunochroma-tography	236 (12.7)	NS	NS	4
Wang 2024 (*65*)	People's Republic of China, Shenzhen	Prospective cohort	Women (age, 18–44 y) attending ANC	939	35–37	Vagina and rectum	Enrichment (Lim broth) and culture (sheep blood agar, MALDI-TOF)	168 (17.9)	NS	NS	5
Xiao 2023 (*66*)	People's Republic of China, Dongguan	Cross-sectional	Women (age, 15–47 y) attending ANC	6,166	35–37	Vagina and rectum	PCR	649 (10.5)	NS	NS	5
Xie 2016 (*67*)	People's Republic of China, Xi'an	Cross-sectional	Women (age range not specified) attending ANC	1,767	32–37	Vagina	Culture (sheep blood agar, latex agglutination)	112 (6.3)	NS	NS	3
Yang 2023 (*68*)	People's Republic of China, Shandong	Retrospective analysis	Women (age, 19–52 y) attending ANC and postpartum	6,892	35–38	Vagina and anus	PCR	465 (6.8)	NS	NS	5
Zhang 2021 (*69*)	People's Republic of China, Jilin	Prospective cohort	Women (age, 19–43 y) attending ANC	275	35–37	Vagina and anus	Culture by Granada TM-T media or blood agar, followed by biochemical test, latex agglutination, and VITEK2	20 (7.3)	NS	Agar doubling dilution method (N = 20); All susceptible to PEN, VAN, LNZ. resistant:	5

Reference	Country, City/State/ Region†	Design	Study population	No. pregnant women tested	Gestational age at time of sampling, wk	Sampling site	GBS detection method‡	Prevalence of women with GBS colonization, no. (%)	Serotyping methods; serotype prevalence (%)	AMR testing methods; AMR prevalence (%)	ROB score
										TET (90); ERY (70); CLI (40); LVX (40); CHL (30)	
Zhou 2024 (*70*)	People's Republic of China, Linyi	Prospective	Women (age range not specified) attending ANC	3,269	35–37	Vagina and anus	Culture (blood agar plates followed by BD Phoenix M50)	231 (7.0)	NS	Kirby–Bauer method (N = 189); All susceptible to PEN, VAN, CFZ, CRO. esistant: ERY (71.2), CLI (77.1), LVX (44.4)	8
Zhu 2019 (*71*)	People's Republic of China, Xiamen	Retrospective analysis	Women (age, <20 to ≥35 y) attending ANC or had preterm delivery	49,908	35–37 or at labor	Lower vagina	Culture (chromogenic agar and morphology assessment)	6,933 (13.9%)	NS	NS	5
Safari 2021 (*72*)	Indonesia, Jakarta	Prospective cohort	Women (age, 17–43 y) attending ANC	177	29–40	Vagina	Enrichment (LIM broth) and culture (StrepB chromID agar plate, sheep blood agar plate, Gram stain, and CAMP test)	53 (29.9)	Multiplex PCR (N = 53); serotype II (30), III (23), Ia and IV each (13), VI (8), Ib and V each (6), NT (2)	Microdilution method (N = 62). All susceptible to VAN, PEN, AMP, CTX, LNZ, DAP. resistant: TET (89), CLI (21), ERY (19), LVX (6).	3
Schwab 2016 (*73*)	Indonesia, Aceh	Prospective cohort	Women (age, 17–42 y) attending ANC	159	14–27	Vagina	Microbiologic culture (not described)	26 (16.4)	NS	NS	8
Esmaon 2024 (*74*)	Malaysia, Kuala Lumpur	Cross-sectional	Women (age, >18 y) attending ANC	573	34–37	Lower vagina and anus	Culture (selective agar plate)	235 (41.0)	NS	NS	7

Reference	Country, City/State/ Region†	Design	Study population	No. pregnant women tested	Gestational age at time of sampling, wk	Sampling site	GBS detection method‡	Prevalence of women with GBS colonization, no. (%)	Serotyping methods; serotype prevalence (%)	AMR testing methods; AMR prevalence (%)	ROB score
Akkaneesermsaeng 2019 (*75*)	Thailand, Songkhla	Cross-sectional	Women (age, >18 y) admitted for labor	505	≥28	Vagina and rectum	Enrichment (LIM broth) and culture (sheep blood agar, biochemical test and latex agglutination)	57 (11.3)	NS	NS	5
Khamching 2017 (*76*)	Thailand, Chiang Mai	Prospective cohort	Women (age, 14–42 y) attending ANC	100	35–37	Lower vagina and rectum	Enrichment (THB with colistin/nalidixic acid broth) and culture (sheep blood agar, biochemical test, and latex agglutination)	19 (19.0)	NS	Method not stated (N = 19);. All susceptible to PEN. resistant: ERY (20), CLI (40).	7
Lower-middle–income countries											
Saha 2017 (77)	Bangladesh, Mirzapur	Cross-sectional	Women (age, <18 to ≥35 y) attending ANC or labor	1,151	≥30	Lower vagina and rectum	Enrichment (THB with nalidixic acid broth) and culture (sheep blood agar, CAMP test, and latex agglutination)	172 (14.9)	Latex agglutination and PCR (N = 172); serotype Ia (40), V (23), II (14), III (12), Ib (1), VI (5), IX (1), IV (1), VII (2), VIII (0)	NS	5
Tarana 2018 (*78*)	Bangladesh, Dhaka	Cross-sectional	Women (age, 15–39 y) attending hospital outpatient clinic	224	28–37	Vagina	PCR	46 (20.5)	NS	Kirby-Bauer modified disk diffusion method (N = 46); All susceptible to PEN, AMP, CTX, VAN. resistant: CLI (10.7), ERY (21.4), CIP (14.3), DOX (35.7), CHL (28.6), GEN (46.4)	5
Kwatra 2024 (*79*)	Bangladesh, Mirzapur	Prospective	Women (age, 18–45 y)	788	≥37	Lower vagina	Culture (CHROMagar) with or without	138 (17.5)	Latex agglutination (N = 788);	NS	8

Reference	Country, City/State/ Region†	Design	Study population	No. pregnant women tested	Gestational age at time of sampling, wk	Sampling site	GBS detection method‡	Prevalence of women with GBS colonization, no. (%)	Serotyping methods; serotype prevalence (%)	AMR testing methods; AMR prevalence (%)	ROB score
			attending ANC			and rectum	enrichment with LIM broth		serotype Ia (45), Ib (1), II (11), III (10), V (30), VI (5)		
Kwatra 2024 (*79*)	India, Vellore	Prospective	Women (age, 18–45 y) attending ANC	850	≥37	Lower vagina and rectum	Culture (CHROMagar) with or without enrichment with LIM broth	174 (20.5)	Latex agglutination (N = 850); serotype Ia (60), Ib (1), II (5), III (5), V (12), VI (7), VII (10)	NS	8
Ajith 2024 (*80*)	India, Kerala	Cross-sectional	Women (age range not specified) attending ANC	120	35–37	Vagina and rectum	Culture (blood agar plate and CAMP test)	0	NS	NS	2
Faby 2024 (*81*)	India, Tamil Nadu	Prospective cohort	Women (age, 19–38 y) attending ANC	150	35–37	Vagina	Enrichment (THB with nalidixic acid broth) and culture (sheep blood agar, biochemical test)	15 (10.0)	NS	NS	6
Geetanjali 2025 (*82*)	India, Puducherry	Case-control	Women (age, 19–39 y) attending ANC	220	35–38	Lower vagina	Culture (sheep blood agar)	8 (3.6)	NS	Method NS (N = 8); susceptible: AMI and GEN (12.5), ERY and AMP (37.5), ERY and PEN and AMP (25), PEN and AMP and CFZ (25)	2
Goel 2020 (*83*)	India, New Delhi	Cross-sectional	Women (age, 18–43 y) attending ANC	450	35–38	Lower vagina and rectum	Enrichment (LIM broth) and culture (sheep blood agar and VITEK2)	15 (3.3)	NS	NS	4
Gogoi 2021 (*84*)	India, Assam	Cross-sectional	Women (age range not specified)	345	35–37	Lower vagina	Enrichment (selective THB media) and culture (sheep	52 (15.1)	Multiplex PCR (N = 19/52 isolates available for	Kirby Bauer disk diffusion method (N = 30); All	6

Reference	Country, City/State/ Region†	Design	Study population	No. pregnant women tested	Gestational age at time of sampling, wk	Sampling site	GBS detection method‡	Prevalence of women with GBS colonization, no. (%)	Serotyping methods; serotype prevalence (%)	AMR testing methods; AMR prevalence (%)	ROB score
			attending ANC				blood agar, biochemical test, and latex agglutination)		serotyping); serotype Ia (42.1), II (15.8), VI (31.6), VII (10.5)	susceptible to AMP, PEN, LVX. resistant: CTX (6.7), ERY (16.7), CLI (16.6), VAN (3.3), CIP (3.3)	
Gurudas 2022 (*85*)	India, Kerala	Cross-sectional	Women (age range not specified) attending ANC and postpartum	966	36–37	Lower and high vagina and rectum	Enrichment (selective THB media) and culture (sheep blood agar, chromogenic agar, and VITEK2)	47 (4.8)	NS	VITEK 2 system (N = 47); susceptible: PEN, AMP, CTX (98), ERY and CLI (85)	6
Hariharan 2022 (*86*)	India, Chennai	Cross-sectional	Women (age, 19–35 y), with or without history of pregnancy complications, attending ANC	150	35–37§	High vagina	Culture (sheep blood agar and HiCHROME selective medium, biochemical test, and latex agglutination)	3 (2.0)	NS	NS	5
Konikkara 2014 (*87*)	India, South Kanara	Cross-sectional	Women (age range not specified) attending ANC	50	35–37	Vagina and rectum	Enrichment (LIM broth) and culture (sheep blood agar, biochemical testing, and latex agglutination)	8 (16.0)	NS	NS	7
Kumar 2023 (*88*)	India, Muzaffarpur	Cross-sectional	Women (age range not specified) attending ANC or undergoing labor	521	>28	High vagina	Enrichment (unspecified media) and culture (StrepB CHROM agar and biochemical testing)	1 (0.2)	NS	NS	4
Nubila 2022 (*89*)	India, Kerala	Cross-sectional	Women (age, 16–40 y) admitted for delivery	250	≥35	Lower vagina and perianal region	Enrichment (THB with nalidixic acid broth) and culture (sheep blood agar, biochemical test,	16 (6.4)	NS	Disk diffusion and D-zone method (N = 16); resistant:	5

Reference	Country, City/State/ Region†	Design	Study population	No. pregnant women tested	Gestational age at time of sampling, wk	Sampling site	GBS detection method‡	Prevalence of women with GBS colonization, no. (%)	Serotyping methods; serotype prevalence (%)	AMR testing methods; AMR prevalence (%)	ROB score
							and latex agglutination)			ERY (12.5), CLI (6.3)	
Warrier 2022 (*90*)	India, South India	Prospective cohort	Women (age, <20 to >30 y) attending ANC	310	≥36	Vagina and rectum	Enrichment (THB with colistin/nalidixic acid broth) and culture (sheep blood agar, biochemical test, and latex agglutination)	40 (12.9)	NS	Kirby-Bauer disk-diffusion method (N = 30); All susceptible to AMP, CTX, PEN, VAN. resistant: CLI (20)	2
Santhanam 2017 (*91*)	India, Vellore	Cross-sectional	Women (age range not specified) attending ANC	305	35–37	Lower vagina and rectum	Enrichment (LIM broth) and culture (sheep blood agar and StrepB CHROM agar plate, morphology assessment, and latex agglutination)	23 (7.6)	NS	NS	8
Khani 2023 (*92*)	Pakistan, Karachi	Prospective cohort	Healthy women and those at risk for GBS (age range not specified) receiving postnatal care	78	¬37	High vagina	NS	23 (29.5)	NS	NS	5
Mumtaz 2020 (*93*)	Pakistan, Lahore	Cross-sectional	Women (age range not specified) attending ANC	200	35	Vagina and rectum	PCR	19 (9.5)	Streptococcal serotyping kit (N = 19); serotype III (36.8), V (21), Ia (15), II (10), VII (5), NT (10).	NS	4
Munir 2016 (*94*)	Pakistan, Lahore	Cross-sectional	Women (age, 20–40 y) attending ANC	200	≥28	Low vagina	Culture (blood agar and biochemical test)	28 (14.0)	NS	NS	1
Navid 2023 (*95*)	Pakistan, Lahore	Descriptive case series	Women (age, 20–45 y) with preterm labor,	150	≥28	Vagina and rectum	Culture (NS)	32 (21.3)	NS	NS	6

Reference	Country, City/State/ Region†	Design	Study population	No. pregnant women tested	Gestational age at time of sampling, wk	Sampling site	GBS detection method‡	Prevalence of women with GBS colonization, no. (%)	Serotyping methods; serotype prevalence (%)	AMR testing methods; AMR prevalence (%)	ROB score
			PPROM, or prolonged ROM								
Wasim 2022 (*96*)	Pakistan, Risalpur	Cross-sectional	Women (age, 18–40 y) attending outpatient clinic	261	28–40	Vagina	NS	48 (18.4)	NS	NS	7
Dilrukshi 2020 (*97*)	Sri Lanka, Western Province	Cross-sectional	Women (age not specified) attending ANC	175	>35	Lower vagina and rectum	Enrichment (THB with nalidixic acid broth) and culture (sheep blood agar, biochemical and latex agglutination) or PCR	45 (25.7)	NS	Disk diffusion and Epsilometer method (N = 45); All isolates susceptible to PEN, AMP, CTX, VAN. resistant: ERY (24.4), CLI (22.2)	3
Dilrukshi 2023 (*98*)	Sri Lanka, Western Province	Cross-sectional	Women (age, 17–44 y) attending ANC	100	>35	Vagina and rectum	Enrichment (THB with nalidixic acid broth) and culture (sheep blood agar, biochemical and latex agglutination) or PCR	18 (18.0)	NS	Disk diffusion and Epsilometer method (N = 18); All isolates susceptible to PEN, AMP, CTX, VAN. resistant: ERY (5.2), CLI (5.2)	6
Nanayakkara 2018 (*99*)	Sri Lanka, Peradeniya	Prospective cohort	Women (age, 17–41 y) admitted for delivery	250	NA	Low vagina	Culture (blood agar) with or without enrichment with THB broth and latex agglutination	37 (14.8)	NS	NS	5
Sapugahawatte 2022 (*100*)	Sri Lanka, Peradeniya	Prospective cohort	Women (age range not specified) admitted to	250	NA	Low vagina	Culture (blood agar) and MALDI-TOF-MS	28 (11.2)	Multiplex PCR (N = 28); serotype VI (17.9), III (25),	Microdilution method (N = 28); All susceptible	5

Reference	Country, City/State/ Region†	Design	Study population	No. pregnant women tested	Gestational age at time of sampling, wk	Sampling site	GBS detection method‡	Prevalence of women with GBS colonization, no. (%)	Serotyping methods; serotype prevalence (%)	AMR testing methods; AMR prevalence (%)	ROB score
			antenatal ward						II (32.1), Ia (10.7), V (10.7), Ib (13.6)	to PEN, OXA, CTX, CBT, VAN, LNZ, CHL, CLI, LIN, CIP, LVX, GAT, MFX. resistant: GEN (100), DOX (56.7), MIN (56.7), TET (58.3), ERY (15).	
Ha 2024 (*101*)	Vietnam, Ho Chi Minh City	Cross-sectional	Women (age range not specified) attending ANC	259	>35	Vagina and rectum	Culture (blood agar) with or without enrichment with THB broth and StrepB CHROM agar plate	66 (25.5)	NS	NS	6
Hanh 2020 (*102*)	Vietnam, Nghe An	Cross-sectional	Women (age range not specified) attending ANC	750	35–37	Vagina	Culture (blood agar and CAMP test)	69 (9.2)	Multiplex PCR (N = 69); serotype III (39.1), V (31.9), Ia (11.6), VI (11.6), Ib (2.9), II (1.5), VII (1.5); Serotypes IV, VIII. and IX were not detected.	NS	8
Nguyen 2025 (*103*)	Vietnam, Hanoi	Cross-sectional	Women (age, 17–43 y) attending ANC	876	35–37	Vagina and rectum	Enrichment (THB with colistin/nalidixic acid broth) and culture (sheep blood agar and StrepB CHROM agar plate, biochemical test)	171 (19.5)	Multiplex PCR (N = 171); serotype Ia (10.5), Ib (10), II (8.8), III (23.4), V (21.6), VI (12.3), VII (0.6), NT (12.3).	NS	8

Reference	Country, City/State/ Region†	Design	Study population	No. pregnant women tested	Gestational age at time of sampling, wk	Sampling site	GBS detection method‡	Prevalence of women with GBS colonization, no. (%)	Serotyping methods; serotype prevalence (%)	AMR testing methods; AMR prevalence (%)	ROB score
Nguyen 2022 (*104*)	Vietnam, Hue	Cross-sectional	Women (age, <18 y to ≥35 y) with aerobic vaginitis attending ANC or labor	50	≥28	Vagina	Culture (blood agar plate and MALDI-TOF MS)	3 (6.0)	NS	NS	6
Tran 2021 (*105*)	Vietnam, Can Tho	Cross-sectional	Women (age, 21–41 y) attending ANC	203	35–37	Vagina and rectum	Culture (microbial growth media, blood agar plate, Gram stain, and CAMP test)	33 (16.3)	NS	Disk diffusion method (N = 33); susceptible: VAN (81.8), CFZ (69.7), ERY (66.7). resistant: AMP (87.9), CLI (63.7), ERY (24.2)	1
VanDu 2021 (*106*)	Vietnam, Hanoi	Cross-sectional	Women (age, 18–54 y) attending ANC	3,863	8–41.2	Vagina and rectum	Enrichment (THB media) and culture (sheep blood agar, biochemical and CAMP tests, and latex agglutination)	310 (8.0)	NS	Kirby-Bauer disk diffusion method, VITEK2, E-test (N = 33–272); All were sensitive to AMP, PEN, CTX, CRO, SYN. resistant: TET (89.66), ERY (76.23), CLI (58.21), CHL (52.38), LVX (28.46)	4

*Amp, ampicillin; ANC, antenatal care; AZI, azithromycin; CAMP, Christie–Atkins–Munch-Peterson; CBT, ceftibuten; CFZ, cefazolin; CHL, chloramphenicol; CIP, ciprofloxacin; CLA, clarithromycin; CLI, clindamycin; CRO, ceftriaxone; CTX, cefotaxime; DAP, daptomycin; DOX, doxycycline; ERY, erythromycin; FEP, cefepime; GAT, gatifloxacin; GEN, gentamicin; LIN, lincomycin; LNZ, linezolid; LVX, levofloxacin; MALDI-TOF MS, matrix-assisted laser desorption/ionization time-of-flight mass spectrometry; MEM, meropenem; MIN, minocycline; MXF, moxifloxacin; NA, not applicable; NS, not stated; NT, not typeable; OXA, oxacillin; PEN, penicillin; PPROM, preterm prelabor rupture of membranes; QUI, quinupristin; SYN, quinupristin/dalfopristin; TEC, teicoplanin; TEL, telithromycin; TET, tetracycline; THB, Todd Hewitt broth; TPROM, prelabor rupture of membranes at term; TSA; tryptone soy agar; VAN, vancomycin.
†City not stated indicates either not reported or included all cities (nationwide study).
‡Biochemical test includes but is not limited to the CAMP test, catalase test, or morphology assessment.
§Any gestational age for high-risk women.

Appendix Table 4. Study risk of bias scores according to Joanna Briggs Institute risk of bias analysis tool with criteria defined in Table 1*

Category	References								
Item no.	Braye 2021	Braye 2019	Chan 2014	Furfaro 2019	Furfaro 2019	McCloskey 2017	Mehta 2025	Moorhead 2019	Pangerl 2022
1	Unclear	Yes	Unclear	Yes	Yes	Yes	Yes	No	Yes
2	Unclear	Unclear	Yes	No	Yes	Yes	Unclear	No	No
3	Yes	Yes	Yes	Unclear	Yes	Yes	Yes	Yes	Yes
4	Unclear	Unclear	Yes	Yes	Yes	Yes	Yes	No	Unclear
5	Yes	Yes	Yes	Yes	Yes	Yes	Yes	No	Yes
6	Unclear	Unclear	Yes	Yes	Yes	Unclear	Unclear	Unclear	Unclear
7	Unclear	Unclear	Yes	Yes	Yes	Unclear	Unclear	Unclear	Unclear
8	Yes	Yes	Yes	Yes	Yes	Yes	Yes	Yes	Yes
Score	3	4	7	6	8	6	5	2	4
Item no.	Thompson 2025	Chan 2023	Fung 2024	Li 2019	Ma 2018	Seto 2019	Yeung 2015	Kawaguchiya 2022	Morita 2014
1	Yes	Yes	Yes	No	Yes	Yes	No	Unclear	Unclear
2	Unclear	Yes	Unclear	Unclear	Unclear	Yes	Yes	Unclear	No
3	Yes	Yes	Yes	Yes	Yes	Yes	No	Yes	Yes
4	Unclear	Unclear	Yes	Yes	Yes	Yes	No	No	No
5	No	No	Yes	Yes	Yes	Yes	Yes	Yes	Yes
6	Unclear	Yes	Unclear	Unclear	Yes	Yes	Yes	No	Yes
7	Unclear	Unclear	Unclear	Yes	Yes	Yes	Yes	Unclear	Yes
8	Yes	Yes	Yes	Yes	Yes	Yes	Yes	No	No
Score	3	5	5	5	7	8	5	2	4
Studies 19–27									
Item no.	Nakano 2024	Shibata 2021	Takayama 2017	Tanaka 2016	Tano 2021	Tanno 2024	Yoshida 2025	Bae 2022	Choi 2021
1	Yes	Yes	Yes	Unclear	Yes	No	Yes	Yes	Yes
2	Unclear	Yes	Unclear	Yes	Yes	Unclear	Yes	Unclear	Yes
3	No	Unclear	Unclear	No	Yes	Yes	Yes	Yes	Unclear
4	No	Yes	Unclear	Yes	Yes	No	Unclear	Unclear	Yes
5	Yes	Yes	Yes	Yes	No	Yes	Yes	Yes	Yes
6	Yes	No	Yes	Yes	No	Yes	Yes	No	Yes
7	Yes	Yes	No	Yes	No	Yes	Unclear	Unclear	Yes
8	Unclear	Yes	Yes	Yes	Yes	Yes	Yes	Yes	No
Score	4	6	4	6	5	5	6	4	6
Studies 28–36									
Item no.	Kim 2018	Lee 2019	Song 2022	Sung 2022	Chang 2017	Cho 2019	Hung 2018	Lai 2021	Lee 2015
1	Yes	No	Unclear	Yes	Yes	Yes	Unclear	No	Yes
2	Unclear	Unclear	Yes	Unclear	Yes	Unclear	No	Yes	No
3	Yes	No	Unclear	Yes	Yes	Yes	Yes	Yes	Yes
4	Yes	No	No	Unclear	Yes	Yes	Yes	Yes	No
5	Yes	Yes	Yes	Yes	No	Yes	Yes	Yes	Yes
6	Yes	Yes	Yes	Yes	Unclear	Unclear	Unclear	Yes	Yes
7	Yes	Yes	Yes	Yes	Unclear	Unclear	Unclear	Yes	No
8	Yes	Yes	Yes	Yes	Yes	Yes	Yes	Yes	Unclear
Score	7	4	5	6	5	5	4	7	4
Studies 37–45									
Item no.	Li 2016	Wang 2022	Chen 2018	Chen 2018	Chen 2019	Chen 2022	Chen 2023	Chen 2024	Dai 2019
1	No	Yes	Yes	Yes	Yes	Yes	Unclear	Unclear	Yes
2	Unclear	Yes	Yes	Yes	Yes	Yes	Yes	Yes	Yes

Category	References								
3	Yes	Yes	Yes	Yes	Yes	No	Yes	Yes	Unclear
4	No	Yes	Yes	Yes	Yes	Yes	Yes	Unclear	Yes
5	Yes	Yes	Yes	Yes	Yes	Yes	Yes	Yes	Yes
6	No	Yes	Yes	Yes	Yes	Unclear	Yes	No	Unclear
7	Yes	Yes	No	Yes	No	Yes	Yes	Unclear	Yes
8	No	Yes	Yes	Yes	Yes	Yes	Yes	Yes	Yes
Score	3	8	7	8	7	6	7	4	6
Studies 46–54									
Item no.	Du 2016	Gao 2021	Ge 2021	Ji 2017	Ji 2019	Jiao 2022	Jin 2025	Lai 2025	Li 2023
1	Yes	Unclear	Yes	Yes	No	Yes	Yes	Unclear	Yes
2	Yes	Yes	No	No	Yes	No	Unclear	Unclear	Unclear
3	Yes	Yes	Yes	Yes	Yes	Yes	Yes	Yes	Yes
4	No	Unclear	Unclear	Yes	Yes	Yes	Yes	No	No
5	Yes	Yes	Yes	No	Yes	Yes	No	Unclear	Yes
6	No	Yes	Unclear	Yes	Unclear	Unclear	No	Yes	Yes
7	Yes	Yes	No	Yes	Yes	Yes	Yes	No	Yes
8	Yes	Yes	Yes	Yes	Yes	Yes	Yes	Yes	Yes
Score	6	6	4	6	6	6	5	3	6
Studies 55–63									
Item no.	Li 2025	Liao 2022	Liu 2025	Liu 2023	Lu 2014	Lu 2024	Ma 2024	Wang 2015	Wang 2018
1	Yes	Unclear	Unclear	Yes	Yes	Unclear	Yes	Unclear	Unclear
2	Unclear	No	Unclear	No	Unclear	Unclear	Yes	No	Yes
3	Yes	No	No	Yes	Yes	Yes	Yes	Yes	Yes
4	Yes	No	Unclear	Yes	Unclear	No	Yes	No	No
5	Yes	Yes	Yes	Yes	Yes	Unclear	Unclear	Yes	Yes
6	Yes	Unclear	Yes	Yes	No	No	Yes	No	Yes
7	Yes	No	No	Yes	Yes	Yes	No	Yes	No
8	Yes	Yes	Yes	Yes	Yes	Yes	Yes	Yes	Yes
Score	7	2	3	7	5	3	6	4	5
Studies 64–72									
Item no.	Wang 2020	Wang 204	Xiao 2023	Xie 2016	Yang 2023	Zhang 2021	Zhou 2024	Zhu 2019	Safari 2021
1	Unclear	Unclear	Yes	Yes	Yes	Unclear	Yes	Yes	Yes
2	Unclear	Unclear	No	Unclear	Yes	Yes	Yes	Unclear	Unclear
3	Yes	Yes	Yes	Yes	Yes	Unclear	Yes	Yes	No
4	No	No	No	No	Yes	No	Yes	Yes	Yes
5	Unclear	Yes	Yes	No	Yes	Yes	Yes	Yes	Yes
6	No	Yes	No	Unclear	No	Yes	Yes	No	Yes
7	No	Yes	Yes	No	Yes	Yes	Yes	No	No
8	Yes	Yes	Yes	Yes	Yes	Yes	Yes	Yes	Yes
Score	2	5	5	3	7	5	8	5	5
Studies 73–81									
Item no.	Schwab 2016	Esamoan 2024	Akkaneeser msaeng 2019	Khamching 2017	Saha 2017	Tarana 2018	Kwatra 2024	Ajith 2024	Faby 2024
1	No	Yes	Yes	Unclear	Yes	No	Yes	Unclear	Unclear
2	Unclear	Yes	Yes	Yes	Yes	Unclear	Yes	Yes	No
3	No	Yes	Yes	Unclear	Yes	Unclear	Yes	No	No
4	Yes	Yes	Yes	Unclear	Yes	No	Yes	No	Unclear
5	Yes	Yes	Yes	Yes	Yes	Yes	Yes	Yes	Yes

Category	References								
6	Unclear	Yes	Yes	Yes	Yes	No	Yes	No	Yes
7	No	Unclear	Yes	Yes	Yes	No	Yes	Unclear	No
8	Yes	Yes	Yes	Yes	Yes	Unclear	Yes	Unclear	Yes
Score	3	7	8	5	8	1	8	2	3
Studies 82–90									
Item no.	Geetanjali 2024	Goel 2020	Gogoi 2021	Gurudas 2022	Hariharan 2022	Konikkara 2024	Kumar 2023	Nubila 2022	Warrier 2022
1	Unclear	Unclear	Yes	Yes	Unclear	Yes	Yes	Unclear	Yes
2	Unclear	Yes	Yes	Yes	Yes	No	Unclear	Yes	Yes
3	Unclear	Unclear	Unclear	Yes	No	No	Unclear	Unclear	Yes
4	Unclear	Yes	Yes	Yes	Unclear	No	Yes	Yes	Yes
5	Yes	Yes	Yes	Yes	Yes	Yes	Yes	Yes	Unclear
6	No	Yes	Yes	Yes	Yes	Yes	Yes	Yes	Yes
7	No	Yes	No	Yes	No	Yes	No	Yes	Yes
8	Yes	Yes	Yes	Yes	Unclear	No	Yes	Yes	Yes
Score	2	6	6	8	3	4	5	6	7
Studies 91–99									
Item no.	Santhanam 2017	Khani 2023	Mumtaz 2020	Munir 2016	Navid 2023	Wasim 2022	Dilrukshi 2020	Dilrukshi 2023	Nanayakkara 2018
1	Unclear	No	Unclear	Yes	No	Unclear	No	No	Yes
2	Unclear	Unclear	No	Yes	Yes	Unclear	No	No	Yes
3	Yes	Unclear	No	Yes	Yes	Yes	No	No	Unclear
4	No	Yes	No	Unclear	Yes	Yes	Yes	No	Yes
5	Yes	Yes	Yes	Yes	Yes	Yes	Yes	Yes	Yes
6	Yes	Unclear	Unclear	No	Unclear	Unclear	No	Yes	Yes
7	Yes	Unclear	Unclear	No	Yes	No	Yes	Yes	No
8	Yes	Yes	No	Yes	Yes	Yes	Unclear	No	Yes
Score	5	3	1	5	6	4	3	3	6
Studies 100–106									
Item no.	Sapugahawatte 2022	Ha 2024	Hanh 2020	Nguyen 2025	Nguyen 2022	Tran 2021	vanDu 2021		
1	Yes	Yes	Yes	Yes	Yes	Yes	Unclear		
2	Yes	Yes	No	Yes	Yes	Yes	Yes		
3	Unclear	Unclear	Yes	Yes	Unclear	Unclear	Yes		
4	No	Yes	No	Yes	Yes	Unclear	Yes		
5	Yes	Yes	Yes	Yes	Yes	Yes	Yes		
6	No	Yes	Unclear	Yes	No	Unclear	Yes		
7	No	Unclear	Yes	Yes	No	Yes	Yes		
8	Unclear	Yes	Yes	Yes	Yes	No	Yes		
Score	3	6	5	8	5	4	7		

*Total score of 6–8 is considered low risk of bias, a score of 3–5 indicates moderate risk, and 0–2 reflects high risk of bias.

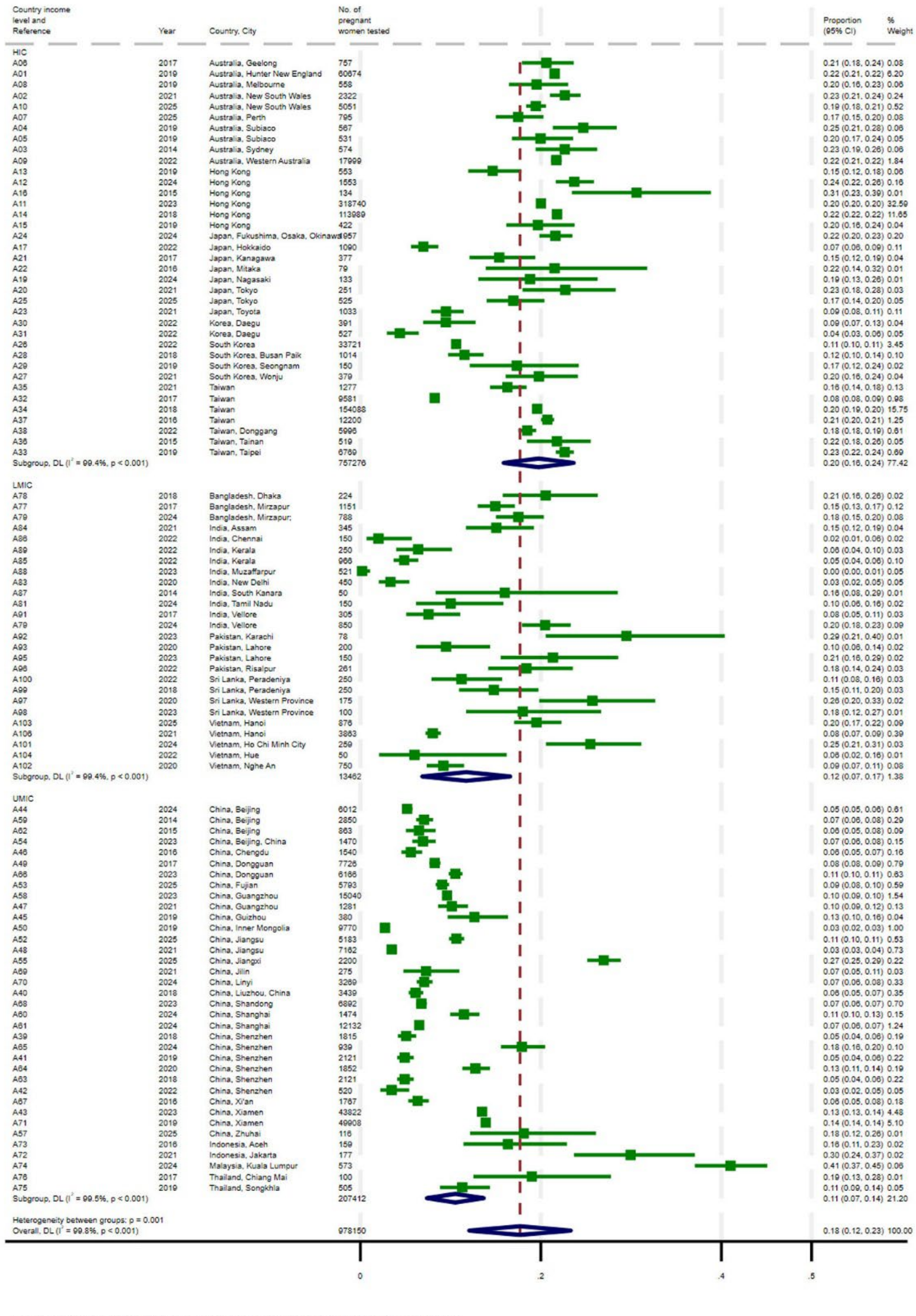

Appendix Figure 1. Forest plot of overall and country income–specific prevalence of GBS colonization among pregnant women in the Asia–Pacific region. Countries are grouped according to World Bank country income status: HIC, high-income countries; UMIC, upper-middle–income countries; LMIC, low-middle–income countries. Red vertical dashed line indicates overall GBS estimates of included studies. Blue diamond indicates overall effect size by country income level. Error bars indicate 95% confidence intervals (CIs). GBS, group B *Streptococcus* disease. DL, DerSimonian-Laird method.

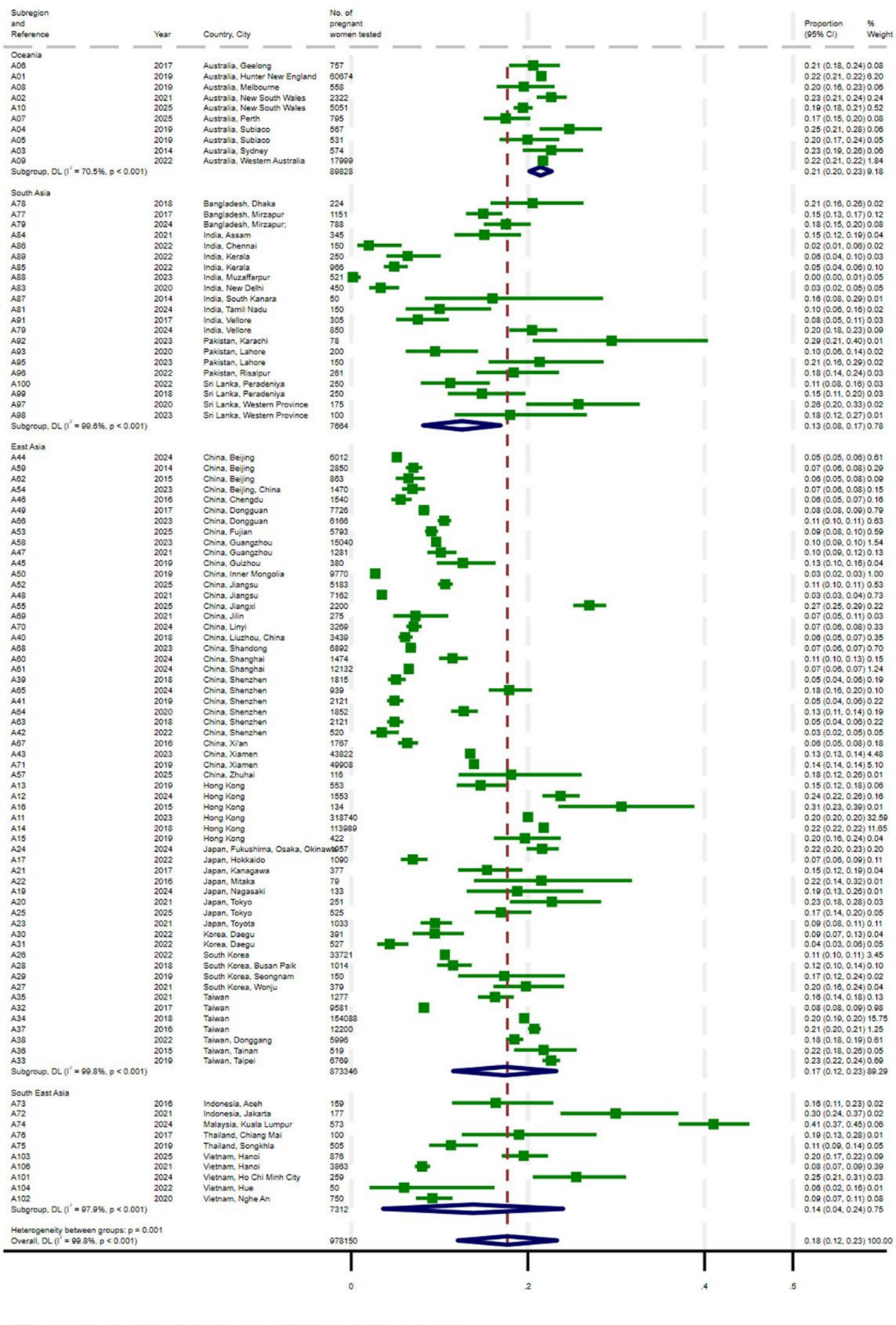

Appendix Figure 2. Forest plot of subregional prevalence of GBS among pregnant women in the Asia–Pacific region. Red vertical dashed line indicates overall GBS estimates of included studies. Blue diamond indicates overall effect size by subregion. Error bars indicate 95% confidence intervals (CIs). GBS, group B *Streptococcus* disease. DL, DerSimonian-Laird method.

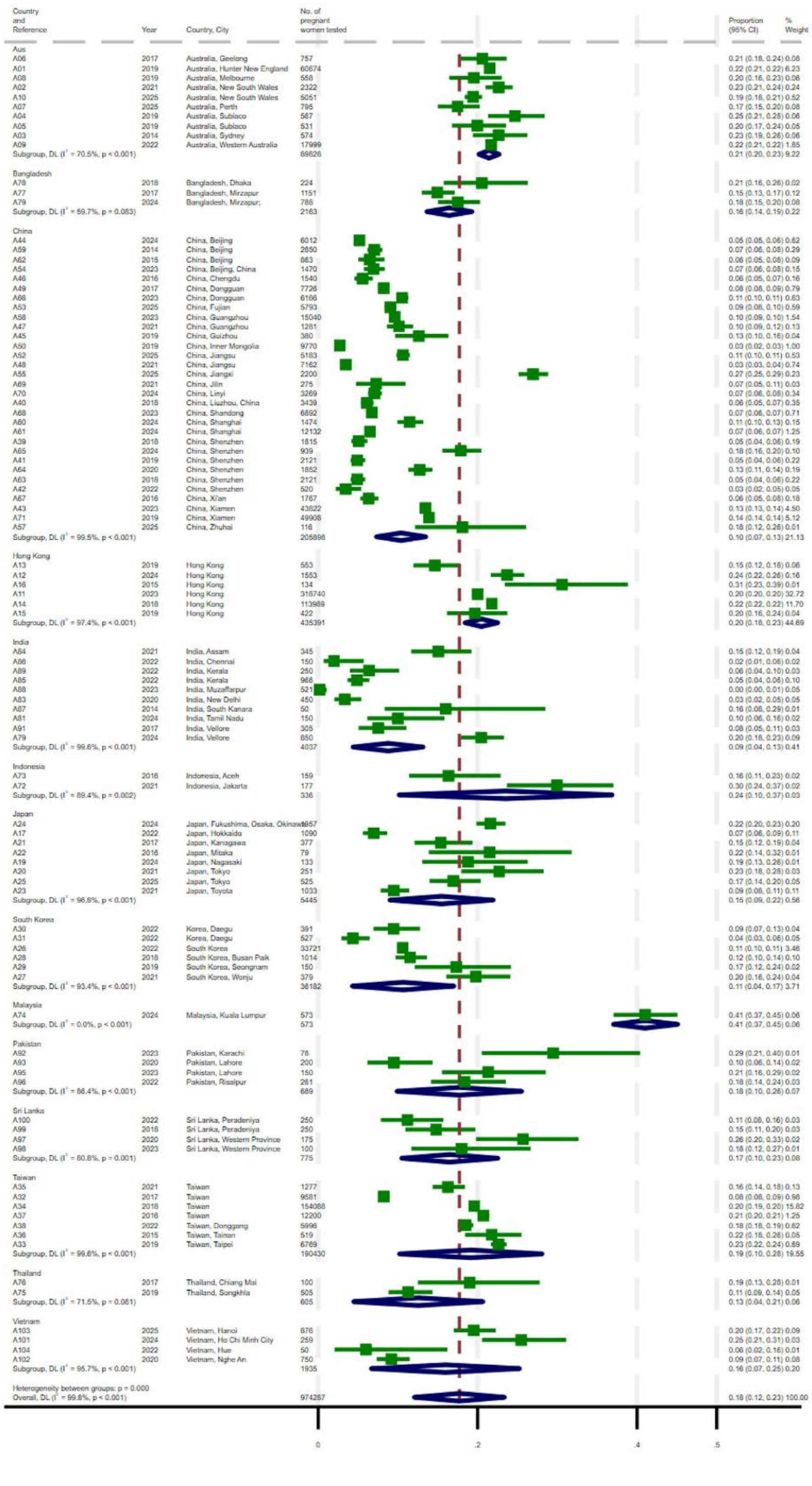

Appendix Figure 3. Forest plot of country-specific prevalence of GBS colonization among pregnant women in the Asia–Pacific region. Red vertical dashed line indicates overall GBS prevalence estimates of included studies. Blue diamond indicates overall effect size by country. Error bars indicate 95% confidence interval (Cis). GBS, group B *Streptococcus* disease. DL, DerSimonian-Laird method.

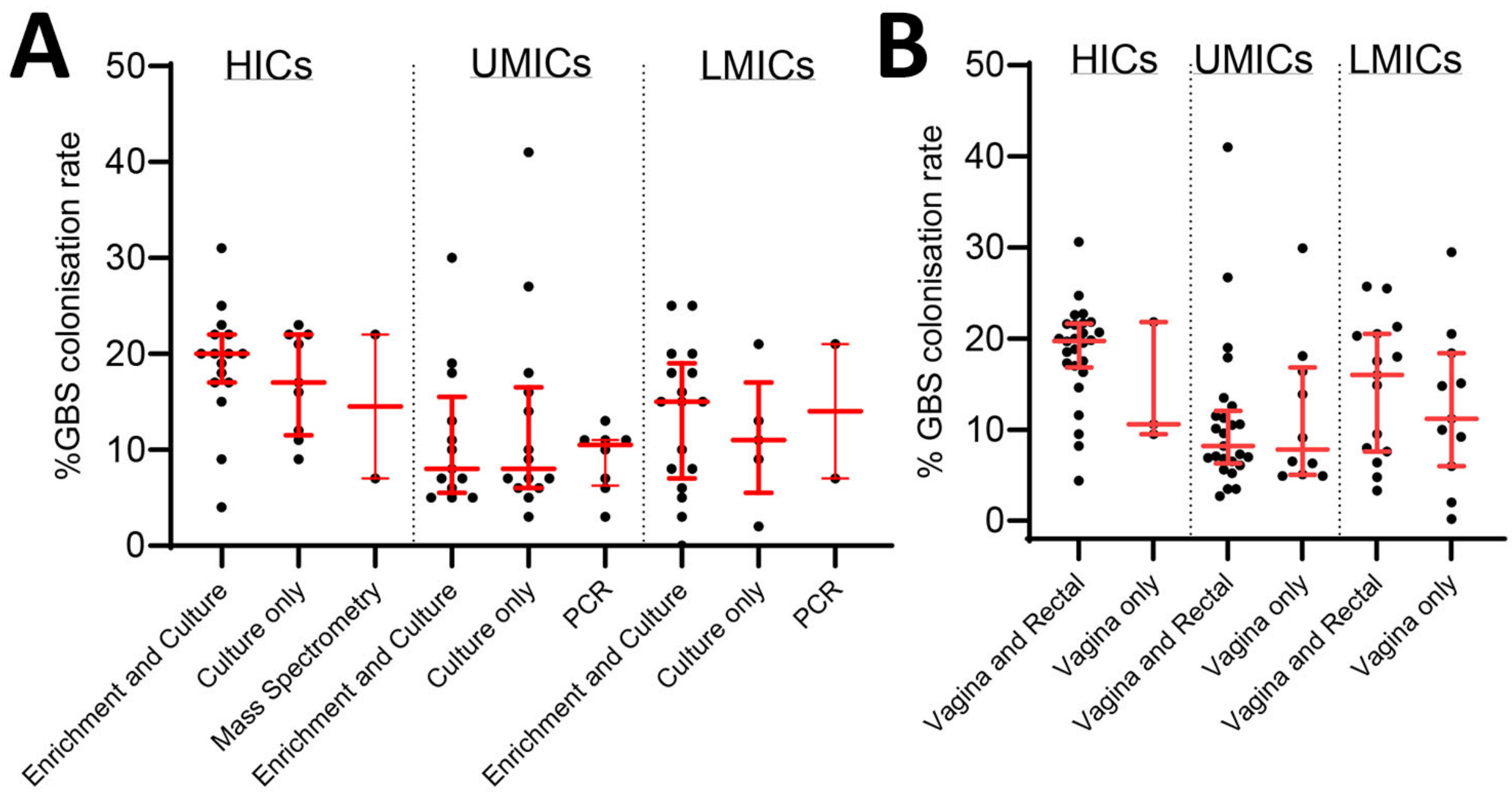

Appendix Figure 4. Subgroup analysis of GBS colonization rates according to A) detection method or B) sampling site grouped by country income level (N = 98 studies). Microbiologic culture on sheep blood, StrepB, or tryptone soy agar plates with or without further identification through biochemical test or latex agglutination test. Enrichment includes LIM broth or supplemented broth culture before microbiologic culture. Fourteen studies did not specify the testing method. One study used immunochromatography (excluded from the graph). No studies included in high-income countries (HICs) used PCR. No studies included in upper-income (UMICs) or lower-income (LMICs) countries used mass spectrometry. Two studies used samples from cervix and vagina (excluded from the graph). Seven studies did not indicate the sampling site.

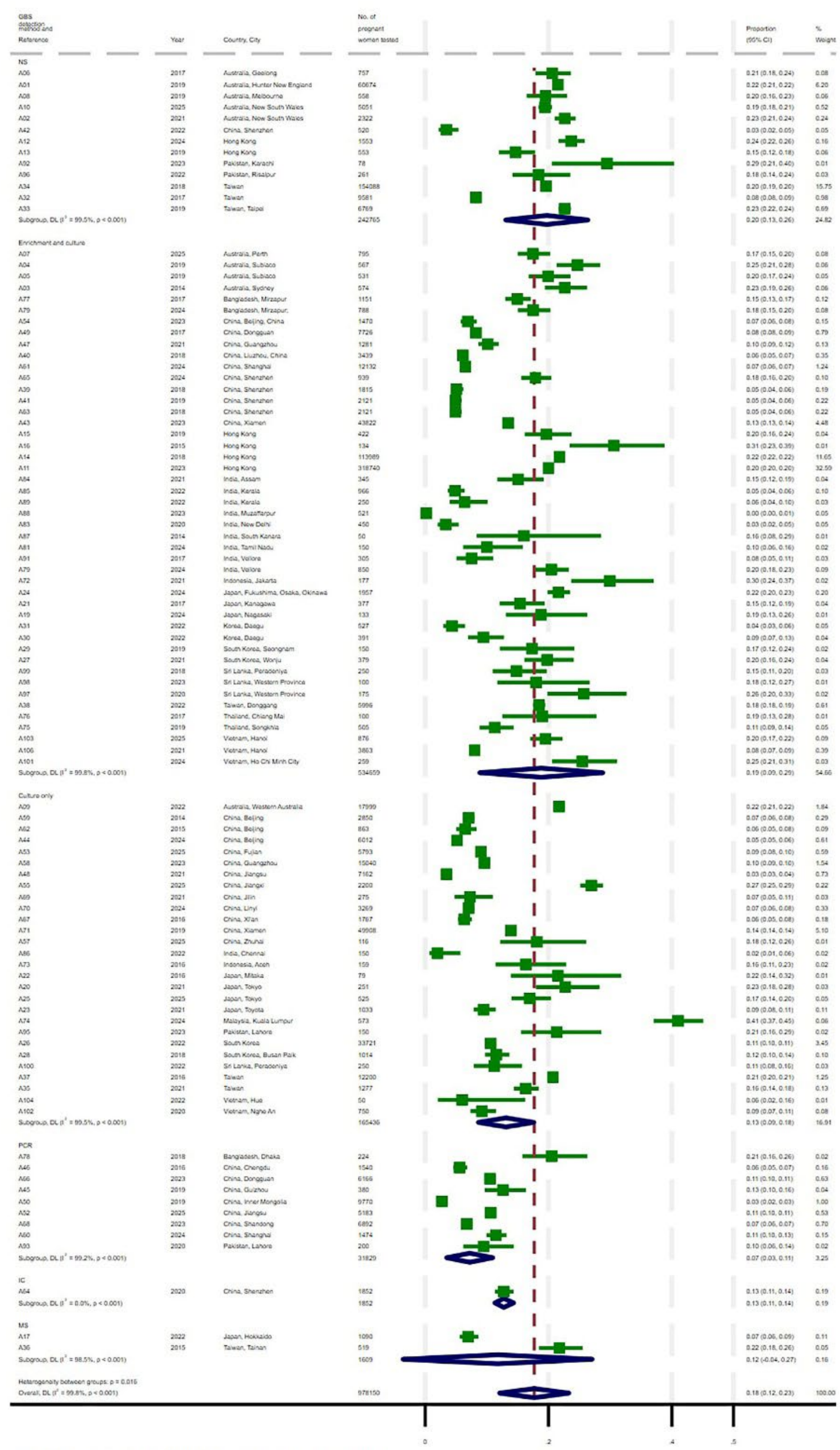

Appendix Figure 5. Forest plot of subgroup analysis of GBS colonization rates according to GBS detection method derived from pooled GBS prevalences using a random-effects inverse variance model weighted by sample size. Culture only: microbiologic culture on sheep blood, StrepB or tryptone soy agar plates with or without further identification through biochemical test or latex agglutination. Enrichment includes LIM broth or supplemented broth culture before microbiologic culture. Fourteen studies did not specify testing method; 1 used immunochromatography. Red vertical dashed line indicates a visual assessment of heterogeneity of the studies. Error bars indicate 95% CIs. GBS, group B *Streptococcus*.

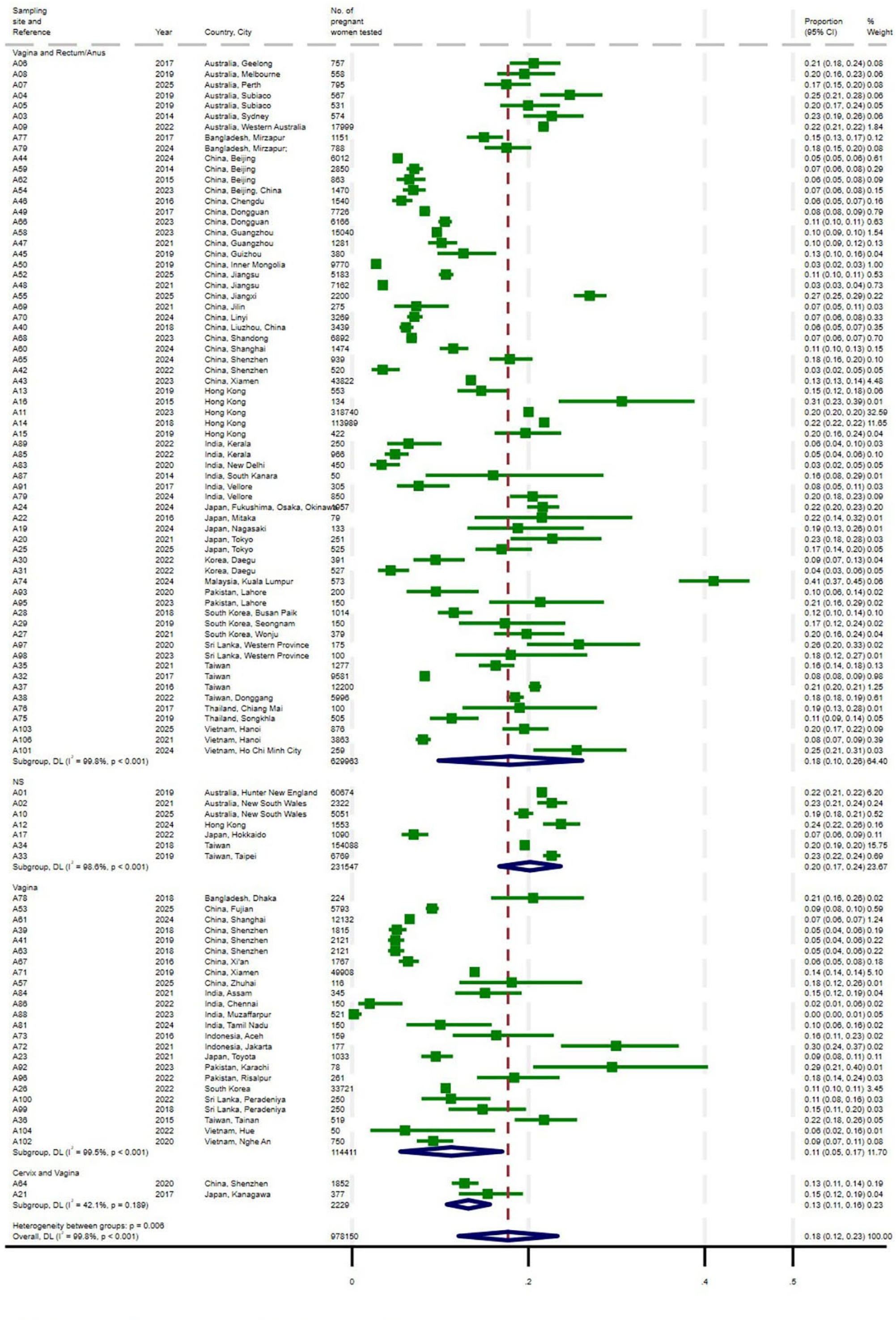

Appendix Figure 6. Forest plot of subgroup analysis according to GBS sampling sites derived from pooled GBS prevalence rates using a random-effects inverse variance model weighted by sample size. GBS, group B *Streptococcus*
